# Benthic-pelagic coupling mediates interactions in Mediterranean mixed fisheries: An ecosystem modeling approach

**DOI:** 10.1371/journal.pone.0210659

**Published:** 2019-01-15

**Authors:** Davide Agnetta, Fabio Badalamenti, Francesco Colloca, Giovanni D’Anna, Manfredi Di Lorenzo, Fabio Fiorentino, Germana Garofalo, Michele Gristina, Lucio Labanchi, Bernardo Patti, Carlo Pipitone, Cosimo Solidoro, Simone Libralato

**Affiliations:** 1 OGS - OCE, Trieste, Italy; 2 CNR-IAS, Castellammare del Golfo (TP), Italy; 3 University of Edinburgh – School of Geosciences, Edinburgh, United Kingdom; 4 SZN, Napoli, Italy; 5 CNR-IRBIM, Mazara del Vallo (TP), Italy; 6 Mably Società Cooperativa, Salerno, Italy; 7 CNR-IAS, Campobello di Mazara (TP), Italy; Aristotle University of Thessaloniki, GREECE

## Abstract

Benthic—pelagic coupling plays a pivotal role in aquatic ecosystems but the effects of fishery driven interactions on its functioning has been largely overlooked. Disentangling the benthic—pelagic links including effects of mixed fisheries, however, needs sketching a whole description of ecosystem interactions using quantitative tools. A holistic food web model has been here developed in order to understand the interplay between the benthic-pelagic coupling and mixed fisheries in a Mediterranean system such as the Strait of Sicily. The reconstruction of the food web required review and integration of a vast set of local and regional biological information from bacteria to large pelagic species that were aggregated into 72 functional groups. Fisheries were described by 18 fleet segments resulting from combination of fishing gears and fishing vessel size. The input-output analysis on the food web of energy pathways allowed identifying effects of biological and fishery components. Results showed that the structure of the Strait of Sicily food web is complex. Similarly to other Mediterranean areas, the food web of the Strait of Sicily encompasses 4.5 trophic levels (TLs) with the highest TLs reached by bluefin tuna, swordfish and large hake and largely impacted by bottom trawling and large longline. Importantly, benthic-pelagic coupling is affected by direct and indirect impacts among groups of species, fleets and fleets-species through the whole trophic spectrum of the food web. Moreover, functional groups able to move on large spatial scales or life history of which is spent between shelf and slope domains play a key role in linking subsystems together and mediate interactions in the Mediterranean mixed fisheries.

## Introduction

Fishing activities targeting benthic and demersal organisms are usually considered unrelated to those targeting pelagic species, and independently managed. In fact, benthic and pelagic domains have been often treated as separate subsystems. However they are not, because of physical [1,2] and biological processes [3] that couple the two domains. As a consequence, fishery driven processes on benthic domain can have a cascading impact on demersal and pelagic domains, and vice-versa. The benthic—pelagic coupling (BPC) plays a pivotal role in aquatic ecosystems by allowing nutrient cycling and energy transfer between the benthic and pelagic domains [4]. In biogeochemistry we often refer to BPC to describe the chemical relationships between nutrient availability and primary producers. Hence, previous studies on BPC have focused mainly on the rules that drive plankton dynamics and sediment biogeochemical processes [5,6]. Little attention has been paid to the role of BPC in a more complex food web especially involving higher trophic levels in concurrency with fisheries interactions.

The release of fecal pellets in the pelagic domains sinking to deeper domains, the occurrence of “marine snow”, the re-suspension of organic material sunk on the bottom [7–9] are examples of processes contributing to BPC, although even more complex and indirect linkages should be considered. Trophic interactions contribute to BPC through movement, feeding habits or behavior of organisms. For example, vertical migrations and horizontal movements of zooplankton, which depend on food availability and predator avoidance mechanisms [10,11] may allow the transport of biomasses, nutrients and energy between coastal and pelagic and between surface and deep domains. Many marine organisms move among habitats during the day [12–14] and their predation and consumption constitute a net transfer of energy between the benthic, demersal and pelagic domains thus allowing for their coupling. Furthermore, ontogenetic diet shifts associated to different habitat preference across life stages represent a net energy flow between different domains. Benthic and pelagic domains are linked by pelagic predators such as tuna and swordfish feeding also on demersal resources [15] while pelagic preys such as sardines and anchovies may feed demersal predators [16]. However, the contribution of trophic interactions to BPC, while highlighted in the literature [3,17] has rarely been quantified in a comprehensive and holistic ecosystemic view.

Fishing is among the most worrisome stressor to BPC across short temporal scales [4,18] and the progressive expansion of fisheries to deeper environments [19,20] has the potential to produce unprecedented disturbances on deep communities with detrimental consequence on ecosystems [21–25]. Indeed, fishing can severely impact on taxa relevant for BPC [26] also through mortality of organisms in their different life stages. Unwanted catches discarded at sea constitute organic matter that sinks to the sea bottom contributing to BPC. Bottom trawling also leads to structurally simpler bottom habitats and impoverished benthic communities [27,28] which may result in fewer chances to exploit energy sources from the water column and leave physical factors to play a major role in structuring BPC. In addition, fishing has been shown to affect trophic interactions, like e.g. with the removal of large predators whose effects propagate through trophic cascade [29,30]. Therefore, the direct and indirect impacts of a mixed fishery might induce important effects on ecosystem dynamics, especially in oligotrophic and semi-enclosed systems such as the Mediterranean Sea [9], and might induce a re-organization of benthic-pelagic fluxes.

The effects of fishing on the functioning of BPC in an ecosystem context, which is a crucial aspect in the Ecosystem-Based Fishery Management (EBFM) [31], has been addressed by several authors generally with the perspective of assessing the multiple effects of trawling on seabed and on traits of benthic organisms [22,32] but the explicit quantification of the contribution of fish and fisheries to BPC needs to be better investigated. In this context ecosystem modelling represents a backbone quantitative way to investigate the role of marine communities and fisheries in BPC. Food web models have been increasingly used to study the effects of fishing and other human activities on the marine ecosystem functioning according to EBFM [33–35], also in the Mediterranean Sea [36]. By integrating large data sets from different sources, such models can represent: i) trophic interactions among the wide biological communities from plankton to top predators, ii) fishing activities with their target and non-target catches, and iii) the effects of fishing and other stressors on all fluxes among functional groups in the food web, including those involved in the BPC and the trade-offs between different fisheries mediated by ecosystem response.

Based on a standard food web ECOPATH model, this paper presents a novel application to the Strait of Sicily (SoS) ecosystem specifically developed for studying the BPC in the Mediterranean Sea. The SoS hosts one of the largest Mediterranean bottom trawl fisheries in terms of fleets, landings and economical incomes [37] and, at the same time, it is characterized by relatively high productivity and biodiversity, wide bathymetric range and habitat complexity [38]. Moreover, a section of SoS fishery targets deep water shrimps by potentially threatening deeper areas [39,40] and possibly influencing BPC also at those depths. In particular, the study allows to identify direct and indirect effects among species, among fleets and between fleets and species. In this way, we used the case of the SoS in order to investigate about the complex nature of the BPC including Mediterranean mixed fisheries. The analysis of the food web model is an attempt to assess how trophic and fishery driven interactions participate directly and indirectly to the BPC in a Mediterranean marine ecosystem.

## Material and methods

### Study area and modelling approach

**Fig 1:**
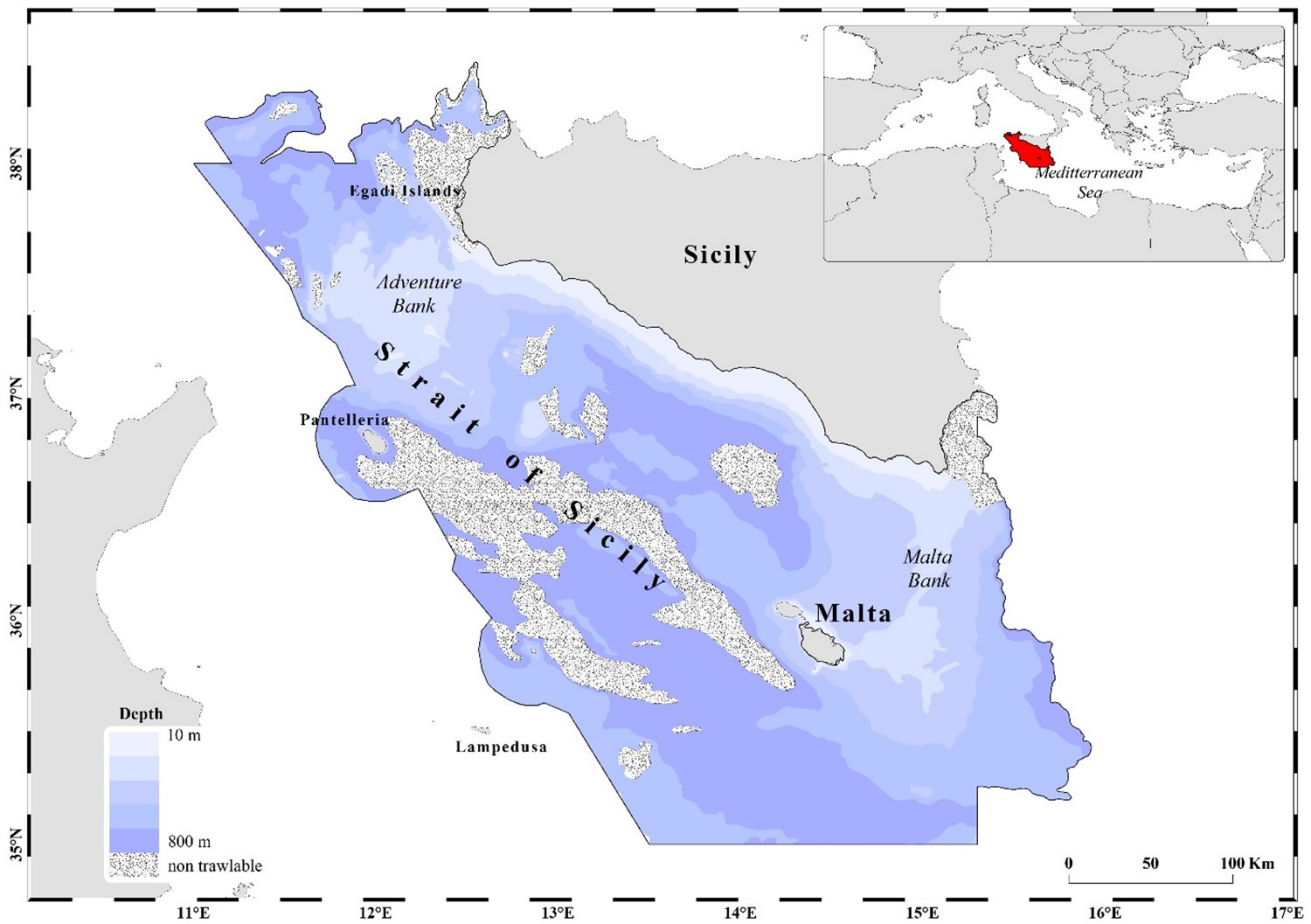
Area of the food web model (about 61,000 km^2^) applied to the Strait of Sicily.

The study area of the food web model coincides with the northern side of the Strait of Sicily, which stretches off the southern Sicily coast and is characterized in its central portion by a narrow continental shelf that separates two wider portions of shelf coinciding with the Adventure Bank in the west and the Malta Bank in the east (Fig 1). The study area has a complex bottom morphology due to the presence of sedimentary and volcanic seamounts [41] that influences the hydrology in the region [42]. The shape of the slope is extremely irregular, incised by several trenches and steep areas. Sea water circulation achieves a two-layer exchange, with an inflow of the Atlantic Ionian Stream flowing eastwards (0–150 m depth) and an undercurrent composed mainly of Levantine Intermediate Water flowing in the opposite direction [43]. Persistent cyclonic vortices around the Adventure and Malta Banks produce upwelling at their center to counterbalance the divergence of surface water [44], whereas frequent wind-induced upwelling events boost primary production in coastal zones [45]. Stable environmental conditions identified around the two banks highly contribute to sustain spawning and nursery areas of commercial species [44,46–49] and hot-spots of biodiversity [50].

The food web model is built using the Ecopath with Ecosim software (EwE v. 6.5, http://www.ecopath.org) [33]. In particular, the Ecopath module was used to integrate a massive amount of environmental, biological and fisheries information in a coherent framework and to describe yearly biomass and flows in the SoS area on the basis of a quantitative mass-balance approach that is widely detailed elsewhere (e.g., [33]) and briefly described in S1 Appendix. We have developed the food web model for the period 2004–2005 considering an area of about 61000 km^2^ at depths ranging between 10 and 800 m, excluding non-trawlable area represented by zones interdicted to fishing, zones deeper than 800 m and hard/rocky substrates (Fig 1). The model area has been defined on the basis of the ecological, bathymetric and fisheries-related information available for the regional area.

### Biological and fisheries input data

The model development required access, review and analysis of data in the SoS for approximately 1400 taxa. Species-specific parameters and dietary data were compiled mainly from publicly available, published and unpublished information, as detailed in S1 Table. In particular, we reviewed and used experimental quantitative information on diet items from the SoS and adjacent areas for more than 200 species.

Fishery-independent biomass density estimates encompassing demersal fishes, cephalopods and other invertebrates were obtained from bottom trawl surveys carried out in spring 2004 and 2005 in the model area within the MEDITS program (Mediterranean International bottom Trawl Surveys) [51]. MEDITS followed a stratified sampling design with proportional allocation of hauls in depth strata (10–50 m, 51–100 m, 101–200 m, 201–500 m, 501–800 m), producing standardized relative biomass estimates per haul and per species (CPUE). Nevertheless, the system equations of EwE are based on absolute biomass, thus MEDITS catches by haul were processed to obtain an average absolute biomass density of each species in the slope and in the shelf portions of the SoS area. MEDITS data by haul were transformed into absolute biomass density data (of the species in the wild, kg km^-2^), by considering species-specific catchabilities. Since the catchability of MEDITS trawl surveys has been seldom studied and is not available for the SoS, we used specific catchability terms derived by the comparison of MEDITS data with independent stock assessments [52] and with surveys with a more efficient gear (beam trawl) from other Mediterranean areas [53] (Tyrrhenian Sea) [24] (Adriatic Sea). Such comparisons (sensu FAO [54]) provided catchability for main species that were compared with published data (e.g. [55]) whenever possible. For European pilchard *Sardina pilchardus* and anchovy *Engraulis encrasicolus*, experimental acoustic surveys carried out in the SoS were taken into account [55,56]. The mega-macrobenthic biomass data set retrieved by the MEDITS was implemented by averaging samples from surveys carried out off the northern Sicily coast as detailed in Romano et al., (2016) [57] (S1 Table). Primary productivity and biomass of phytoplankton, zooplankton, and the concentration of suspended detritus were estimated by averaging the results obtained by the COPERNICUS MedMFC products from the study area [58]. Overall, density estimates for more than 800 taxa were obtained.

For highly migratory species such as bluefin tuna *Thunnus thynnus* and swordfish *Xiphias gladius* we have used the average biomass estimates provided by stock assessments in the Mediterranean Sea [59] taking into account that both species spend a large part of their lifetime outside the SoS area. More specifically, considering the bluefin tuna migratory patterns and assuming a residing time in the modelled area of 4 months year^-1^ [60,61], only 30% of food consumption was considered to occur within the model area (and 70% was set as “import” fraction in the diet). In order to balance the relevant swordfish catches instead, an immigration rate of 0.025 t km^-2^ y^-1^ was considered as the minimum flow to assure mass-balance.

Fishery landings by species and gear in the SoS during 2005 were drawn from the national monitoring of commercial fleets within the European Data Collection Regulation (IREPA; www.irepa.org). Data were aggregated by 18 fleet segments resulting from a combination of fishing gears (i.e., trawlers, purse-seiners, long-liners, netters, etc.) and 3 vessel size classes based on the length overall (LOA): class 1, LOA <12m; class 2, LOA 12-24m; class 3, LOA >24m, bottom otter trawl were also distinguished into categories according to main target species (Table 1). Since the fishing activity of larger bottom trawler targeting deep water species (i.e. deep-water rose shrimp *Parapenaeus longirostris* and giant red shrimp *Aristaeomorpha foliacea*) span over a space larger than the model area [62,63], only 50% of their catches were retained inside the model area (i.e. fleet 18, Table 1) [62]. Empirical discard ratio for commercial species and invertebrates by species and by fleet was drawn from project reports on studies carried out in the SoS [63–67]. For all the other species whose data were not available, it was considered a discard ratio from nearby Mediterranean areas [68–70].

**Table 1:** List of the fleet segments considered by the combination of vessel size (i.e. length overall = LOA) and fishing gear.

N°	LOA	fishing gear	fleet segment
1	1	setgill and trammel net demersal fish	1.GNS
2	1	set and drifting longline	1.LLD
3	1	trolling	1.LTL
4	1	hand-pole cephalopods and finfish	1.LH
5	1	mixed	1.MIS
6	1	purse and boat seine	1.PS
7	2	pots and traps demersal and small pelagic fish	2.FPO
8	2	setgill and trammel net demersal fish	2.GNS
9	2	set and drifting longline	2.LLD
10	2	mixed	2.MIS
11	2	bottom otter trawl_demersal fish	2.OTB_D
12	2	bottom otter trawl_deep water species	2.OTB_DWS
13	2	bottom otter trawl_mixed demersal and deep water species	2.OTB_MDD
14	2	mid water otter trawl_mixed demersal and pelagic species	2.OTM
15	2	pelagic pair trawl_small pelagic fish	2.PTM
16	2	purse and boat seine	2.PS
17	3	mid water otter trawl_mixed demersal and pelagic species	3.OTM
18	3	bottom otter trawl_mixed demersal and deep water species	3.OTB_MDDW

LOA1 = vessel size <12m, LOA2 = 12-24m; LOA3 = >24m.

The definitions of fish functional groups (FGs) were based on a first assessment of diet similarities and life history parameters of involved species using multivariate analysis techniques in order to cluster species with similar diets, growth and mortality rate. FGs definitions were then refined on the basis of expert opinion in order to better describe ecological and fishing features of the SoS. Commercially important species were considered as single FGs, with the red mullet and the European hake represented in 4 and 5 size classes, respectively. Moreover, several FGs were split into shelf and slope components and each group labeled as benthic, demersal or pelagic (Table 2) in order to better represent the BPC. In this way, the starting list of 1400 taxa (from bacteria to large pelagic species) was reduced to 69 living FGs and 3 non-living organic matter compartments.

**Table 2:** Summary description of 72 biological functional groups.

N°	Dom	Name	FG
1	p	Seabirds	SB
2	p	Marine mammals	MM
3	p	Sea turtles	TUR
4	p	Sword fish	XIP
5	p	Bluefin tuna	THU
6	p	Large pelagic fish	LPL
7	p	Medium pelagic fish	MPL
8	p	Other small pelagic fish	SPL
9	d	European hake<6 cm	HAK0
10	d	European hake 6–12 cm	HAK1
11	d	European hake 12.1–22.0 cm	HAK2
12	d	European hake 22.1–41.0 cm	HAK3
13	d	European hake >41.0 cm	HAK4
14	d	Red mullet<8 cm	MUL0
15	d	Red mullet 8–12 cm	MUL1
16	d	Red mullet 12.1–17 cm	MUL2
17	d	Red mullet>17 cm	MUL3
18	d	Horse meckerel	TRA
19	d	Pandora	PAG
20	d	Demersal fish (slope)	DFS
21	d	Demersal fish crustacean feeders (shelf)	DFH
22	d	Demersal fish mixed food (shelf)	DSM
23	d	Demersal fish piscivorous (shelf)	DSP
24	d	Demersal fish rocky (shelf)	DSR
25	d	Mesopelagic fish crustacean feeders (slope)	MSC
26	d	Mesopelagic fish jelly feeders (slope)	MSG
27	d	Mesopelagic fish piscivorous (slope)	MSP
28	d	Rays and skates (shelf)	RSH
29	d	Rays and skates (slope)	RSS
30	d	Sharks (shelf)	SSH
31	d	Sharks (slope)	SSS
32	p	European anchovy	ENG
33	p	European pilchard	SAR
34	p	Epipelagic fish	EPI
35	d	Cephalopods benthic (shelf)	CEBH
36	d	Cephalopods benthic (slope)	CEBS
37	d	Cephalopods pelagic (shelf)	CEPH
38	d	Cephalopods pelagic (slope)	CEPS
39	d	Decapods natant (slope)	DNS
40	d	Decapods natant (shelf)	DNH
41	d	Decapods reptant (slope)	DRS
42	d	Decapods reptant (shelf)	DRH
43	d	Giant red shrimp	ARF
44	d	Deep water rose shrimp	PWL
45	b	Suprabenthos	SUP
46	b	macrobenthos omnivore	O
47	b	macrobenthos filter-feeder	FF
48	b	macrobenthos deposit-feeder	DF
49	b	macrobenthos carnivore	C
50	b	macrobenthos parasite	PAR
51	b	macrobenthos scavenger	SCA
52	b	macrobenthos herbivore	H
53	b	macrobenthos grazer	GRA
54	b	macrobenthos suspension-feeder	SF
55	b	macrobenthos particulate-feeder	PF
56	b	Meiobenthos	BO
57	p	Euphausiacea	EUP
58	p	Gelatinous zooplankton	ZG
59	p	Large zooplankton	ZL
60	p	Mesozooplankton	ZM
61	p	Microzooplankton	ZS
62	p	Pelagic bacteria	PB
63	b	Sediment bacteria	BB
64	p	Pico-phytoplankton	PS
65	p	Dinoflagellates	DFL
66	p	Diatom	PL
67	b	Microphytobenthos	MB
68	b	Seagrass	SG
69	b	Macroalgae	MA
70	b	Detritus Carrion	DC
71	d	Suspended Particulate Organic Matter	SPOM
72	b	Benthic Detritus	BD

Dom = domain: p = pelagic, d = demersal, b = benthic. FG = functional group.

Biomass and catches (landings plus discards) by FG were obtained by summing over the species belonging to each group. Other input parameters (i.e., production per unit of biomass, P/B and consumption per unit biomass, Q/B estimated with empirical parameters) and diet data for each FG were obtained as a weighted average of the values for the species in that group (S1 Table), with the proportion of local species biomass within the group used as the weighting factor [71].

### Analysis of interactions in the BPC context

The basic features of the food web were defined by ecological indices such as Trophic Level (TL), Primary production on Respiration (PP/R) and Primary Production on Biomass (PP/B). The Omnivory Index (OI) and the System Omnivory Index (SOI) were calculated to quantify the width of the trophic spectrum for each FG and as a measure of food web complexity and interconnection, respectively [72].

In order to evaluate the role of all food web components on the BPC we calculated fluxes and impacts among FGs, among fleets and between FGs and fleets. The fluxes are a direct output of the model and were used to determine the strength of direct interactions (i.e., amount of consumption) among FGs and across the three domains considered (i.e., benthic, demersal and pelagic). Direct fishing effects were quantified by i) the exploitation rate E (E = F/Z, where F is the annual fishing mortality and Z is total annual mortality) for each FG belonging to a certain domain, and ii) the cumulative exploitation rate (CumE) for any fleet segment. CumE was calculated (1) in order to compare exploitation across fleets and consequently to visualize which domain was more impacted.

$$CumE_{fleet}=\sum_{i=1}^{n}E_{FGi}$$ (1)

The application of the input-output analysis on the web of flows i.e., the Mixed Trophic Impact analysis (MTI) [73] allowed to quantify the direct and indirect impacts among all biological FGs and fleets. The squared matrix MTI represents the impact of each FG (rows: impacting groups) on any other group of the web (columns: impacted groups). The sum of the MTI elements in the rows allows to obtain the overall cumulative impact (ɛ_i_) produced by a component (biological group or fleet) *i* on the whole food web [74], while the sum by columns allows to obtain the cumulative impact suffered by a component *i*.

Therefore, in order to assess the impact of each FG on BPC, we calculated the portion of cumulative overall impact both positive and negative of a FG on the FGs belonging to the other domains (e.g., FG__demersal_ on FGs__benthic and pelagic_). Finally, we explored the overall effects of fisheries across FGs and fleet segments respectively and the main cascades relevant for BPC.

## Results

### Model validation

**Fig 2:**
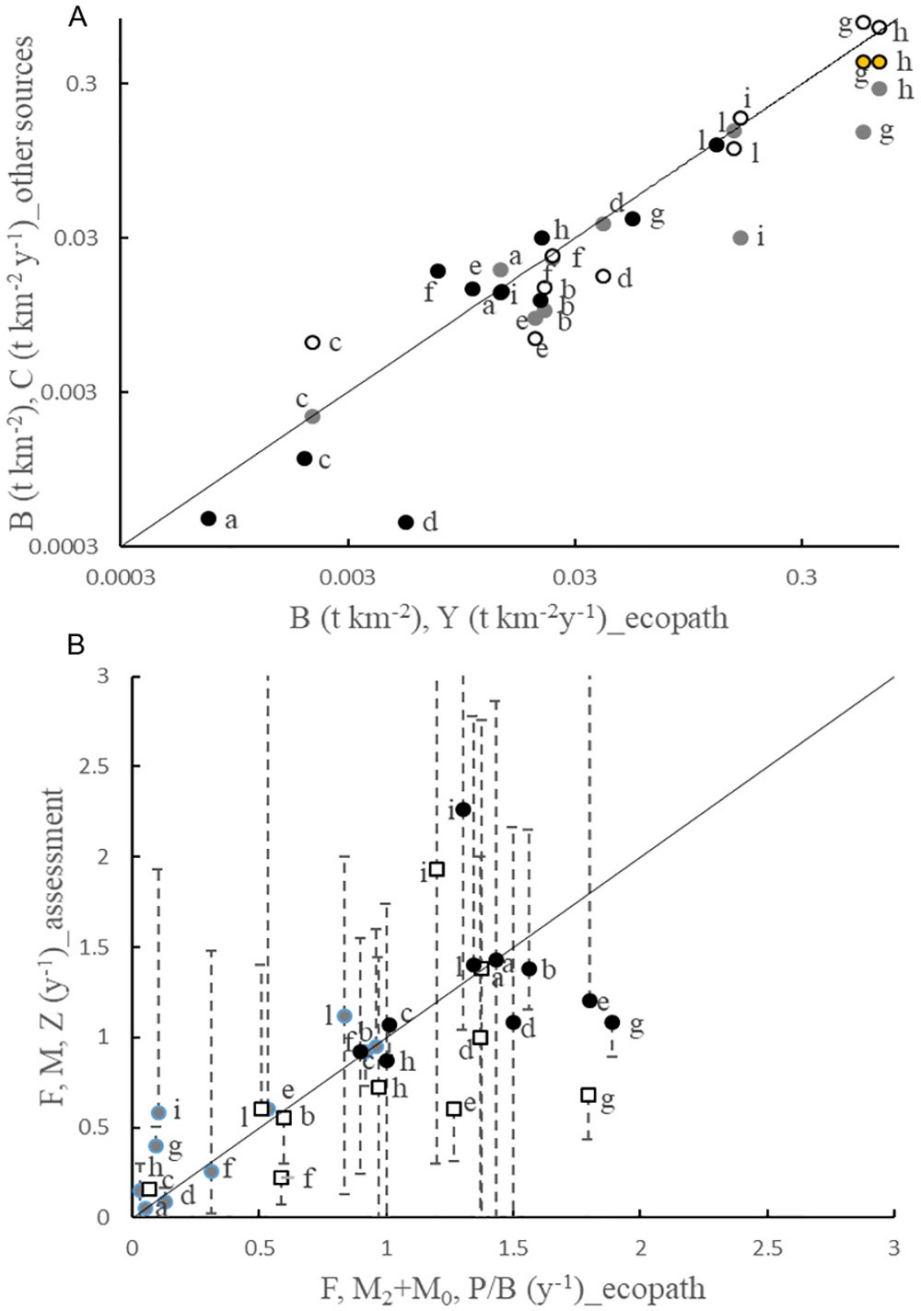
Comparison of variables and parameters for species with detailed stock assessments. (A) Comparison of biomass and catches used in the Ecopath model (x-axis) and other sources of information (y-axis). Biomass (t km^-2^): gray = Ecopath vs stock assessment, white = Ecopath vs MEDITS corrected for catchability, orange = Ecopath vs acoustic-survey only for ENG (g) and SAR (h). Catches (t km^-2^ y^-1^): black = Ecopath vs stock assessment. (B) Comparison of annual mortality rates (y^-1^) used in the Ecopath model and stock assessments. Whiskers indicate MAX and MIN used in stock assessments for different age classes corresponding to Ecopath stanzas. Gray dot = Fishing mortality (F), white square = natural mortality (M) or sum of predation and other mortality (M_2_+M_0_), black dot = total mortality (P/B or Z). a = HAK2, b = HAK3, c = HAK4, d = MUL2, e = MUL3, f = PAG, g = ENG, h = SAR, i = ARF, l = PWL.

Biomass and catches used in the Ecopath model resulted highly correlated (R^2^ = 0.75, F_1,28_ = 29.88, p<0.001) to those from stock assessment and other sources (Fig 2A). Some discrepancies appeared for biomass of anchovy (g) and Giant red shrimp (i) and for catches of red mullet at age class two (d). In particular, for some species such as *Aristaeomorpha foliacea* (i) and *Engraulis encrasicolus* (g) the differences in the areas to which stock assessments and Ecopath refer might explain the discrepancies in the biomasses. Higher catches of red mullet in Ecopath than in assessment models could be instead attributed to higher discards represented in the former. Ecopath mortality rates (Fig 2B) fall in the range of estimates available from stock assessments and were sufficiently correlated (R^2^ = 0.52, F_1,30_ = 90.61, p<0.001). Nevertheless, the comparison highlighted that the integration of data provided by Ecopath results in a general underestimation of fishing mortalities and overestimation of natural mortalities with respect to stock assessments. Globally, variables and parameters used in our model resulted reconciled.

### Structure of SoS food web and direct interactions

**Fig 3:**
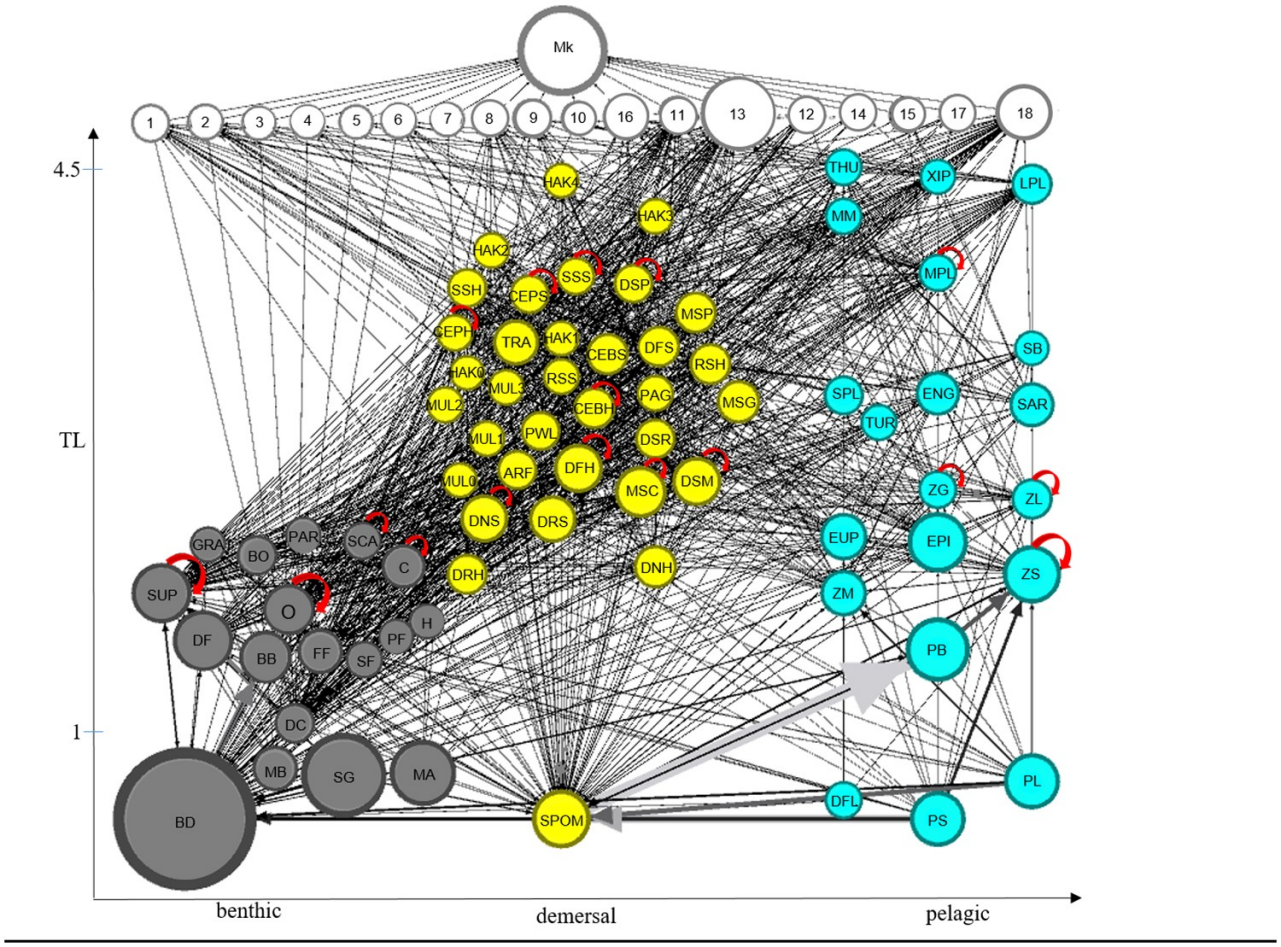
Flow diagram of the food web. Functional groups (nodes) by trophic levels (TL, y-axis) and by benthic (gray), demersal (yellow) and pelagic (cyan) domains (x-axis). White nodes represent fishing activities and the market (Mk). Links width are proportional to flow intensity, i.e., to annual food consumption rates for FG (>5^^-6^ t km^−2^ year^−1^), to catches for fleets and to landings for the market. Node radius is proportional to the square root of FG biomass, total catch of fleets and total landings for the market. Gray arrows indicate higher fluxes. Red arrows are loops (cannibalism).

According to the model, the food web of the Strait of Sicily encompasses 4.5 trophic levels (TLs, Fig 3). The highest TLs were reached by bluefin tuna (THU = 4.55 TL), swordfish (XIP = 4.51) and large hake (HAK4 = 4.48), immediately followed by FGs large pelagic fish (LPL = 4.46) and marine mammals (MM = 4.36). The remaining FGs had TL ranging between 4.31 and 2.87 for fish, and between 2.83 and 2 for macro-benthos and bacteria. Low TL groups had generally higher biomass than those with higher TL thus determining an overall pyramid structure with vertex up. PP/R and PP/B indicated a quite mature ecosystem with a similar contribution of total production (1561 t km^-2^ year^-1^) and consumption (1599 t km^-2^ year^-1^) to the total flows in the system (total system throughput, TST). Total respiratory and detritus flows corresponded to 962 and 967 t km^-2^ year^-1^, respectively.

Globally, FGs resulted well interconnected as shown by SOI = 0.30 and by the fact that more than 50% of total consumer FGs showed a relatively high variability of feeding across trophic levels (OI>0.3).

**Fig 4:**
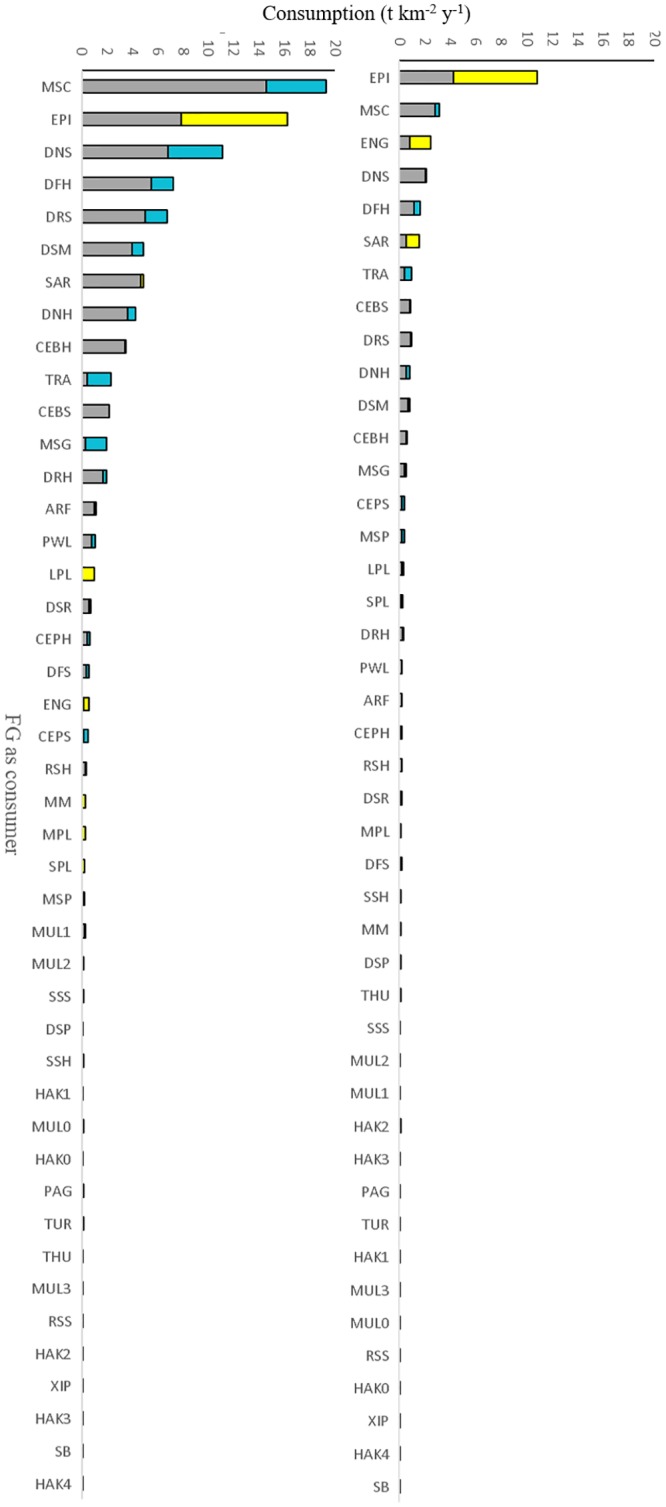
BPC by flux (consumption) of functional groups (FG) acting as consumers (left) and as sources (right) on the 2 other domains they do not belong to. Pelagic (cyan), demersal (yellow), benthic (gray), lower trophic level groups are excluded.

The bulk of consumption fluxes (~95%, 1500 t km^-2^ years^-1^) was exchanged by lower “taxonomic” groups (i.e. macro-benthos, zooplankton, euphausiids and bacteria) across the benthic and pelagic domains (Fig 3). The remaining consumption flux (~ 5%) was determined by all other FGs, with epipelagic fish (EPI) and mesopelagic fish crustacean feeders (MSC) having the most relevant flows for BPC both as consumers and sources (Fig 4). Many other FGs (e.g., horse mackerel *Trachurus spp*., TRA and sardine, SAR), contributed to a less extent to the consumption fluxes linked to BPC (Figs 3 and 4) although they had high biomass and were predators and preys across benthic and pelagic domains as well.

**Fig 5:**
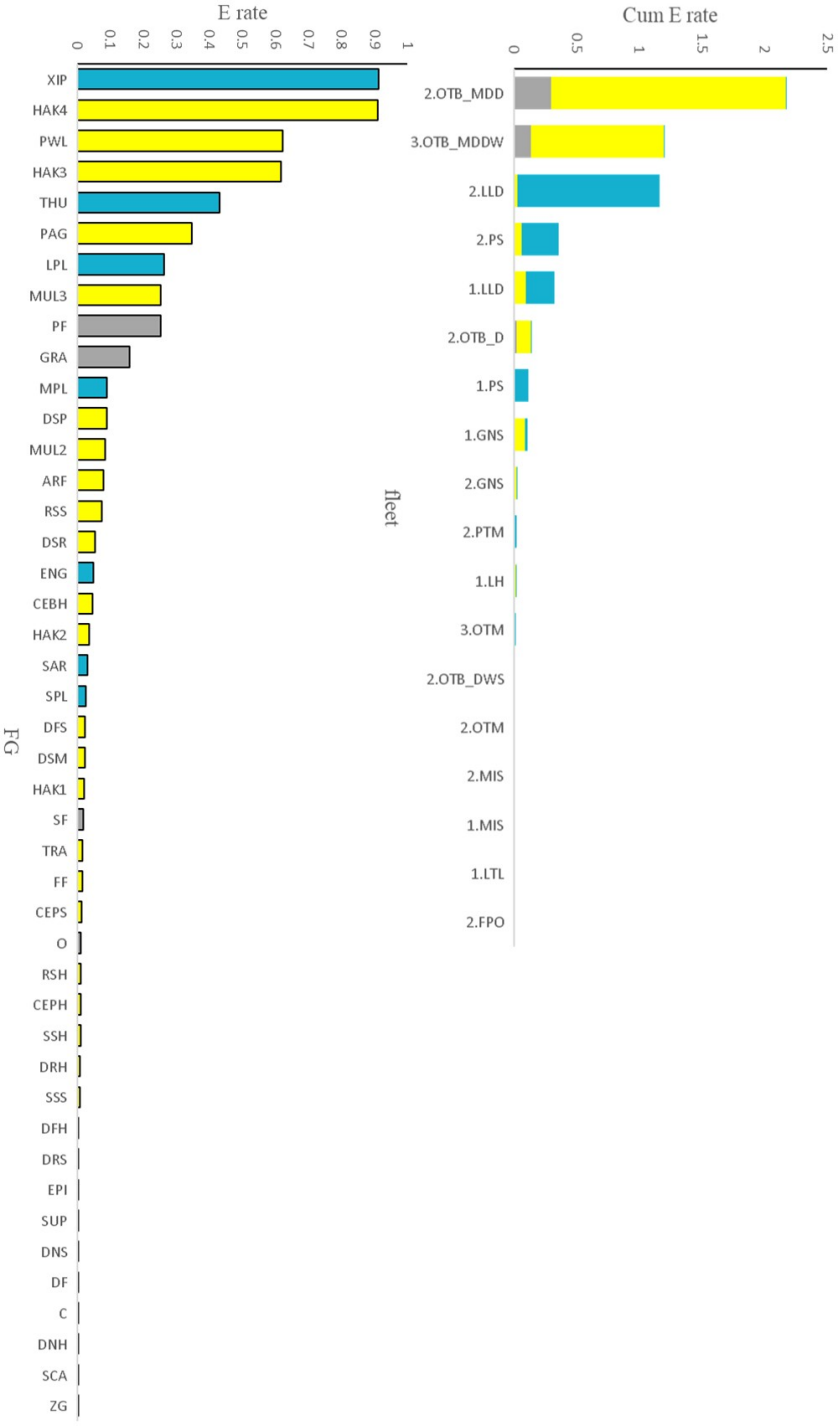
Direct effects of fishing on the 3 domains: Exploitation rate (E) by FGs (left) and cumulative exploitation rate (Cum E) by fleet (right). FGs belonging to pelagic (cyan), demersal (yellow) and benthic (gray) domain. Details of fleets and FGs in Tables 1–2.

The SoS fishing fleet segments, which target resources from shelf and slope, directly impact all three domains with a prevalence of impacts on the demersal and pelagic domains (Fig 5). Bottom trawlers of LOA classes 2 and 3 showed higher values of cumulative exploitation rate followed by long-liners and purse seiners of LOA class 2 (Fig 5). As a consequence, the most highly exploited groups were swordfish (XIP), large European hake (HAK3, HAK4), deep-water rose shrimp (PWL) and bluefin tuna (THU). Pandora *Pagellus erythrinus* (PAG), large red mullet (MUL3) and large pelagic fishes (LPL) also suffered high exploitation rates (Fig 5). Globally, the demersal domain resulted the most directly impacted by fishing activities.

### Overall trophic relationships and FGs relevant for BPC

**Fig 6:**
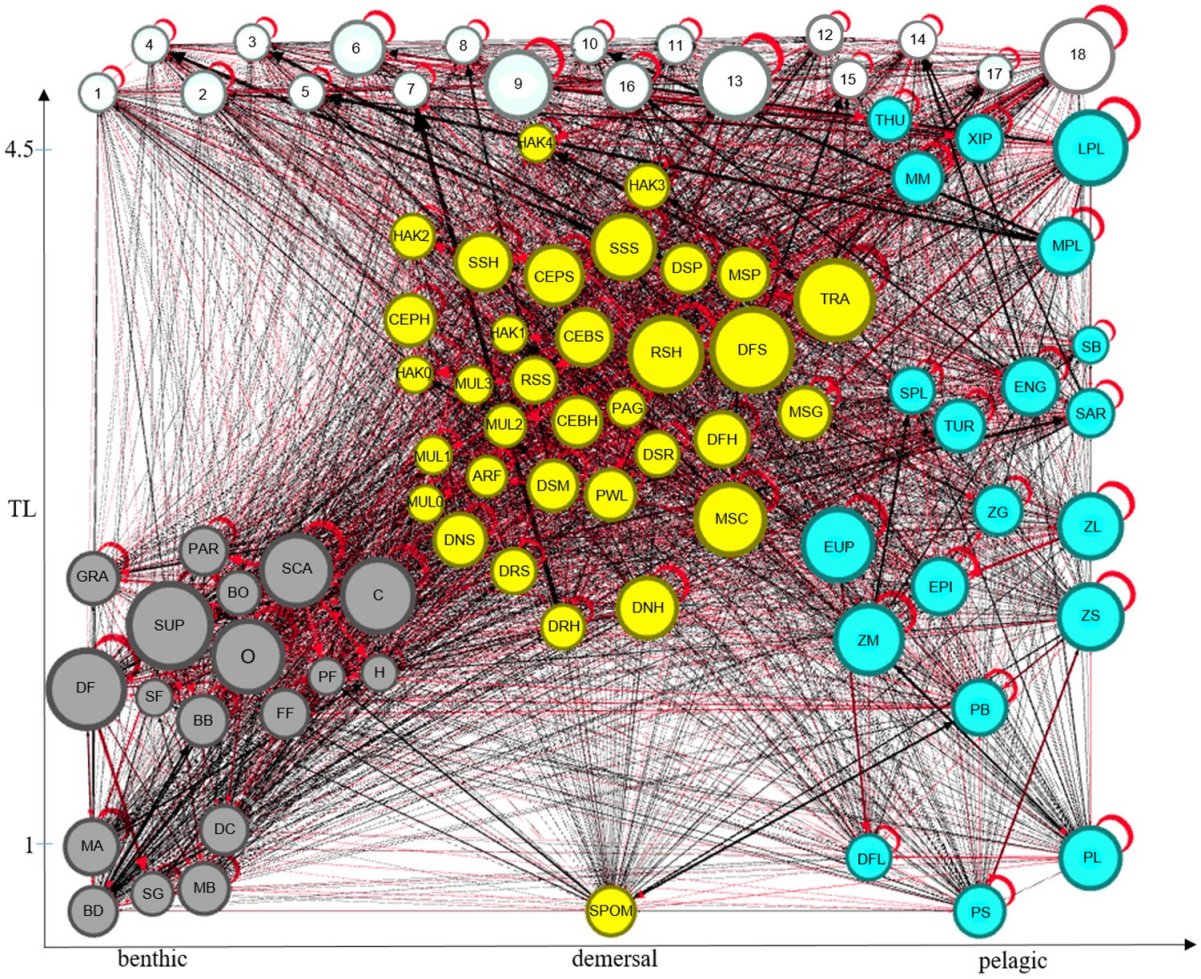
Direct and indirect effects in the SoS food web. Black arrows are positive effects, red arrows are negative effects. Arrow thickness is proportional to MTI (min = 0.03, max = 0.95). The 72 FGs are distinguished into main domain (benthic, demersal, pelagic) and by TL. Their overall cumulative effect in the web is proportional to the dimension of the circle.

**Fig 7:**
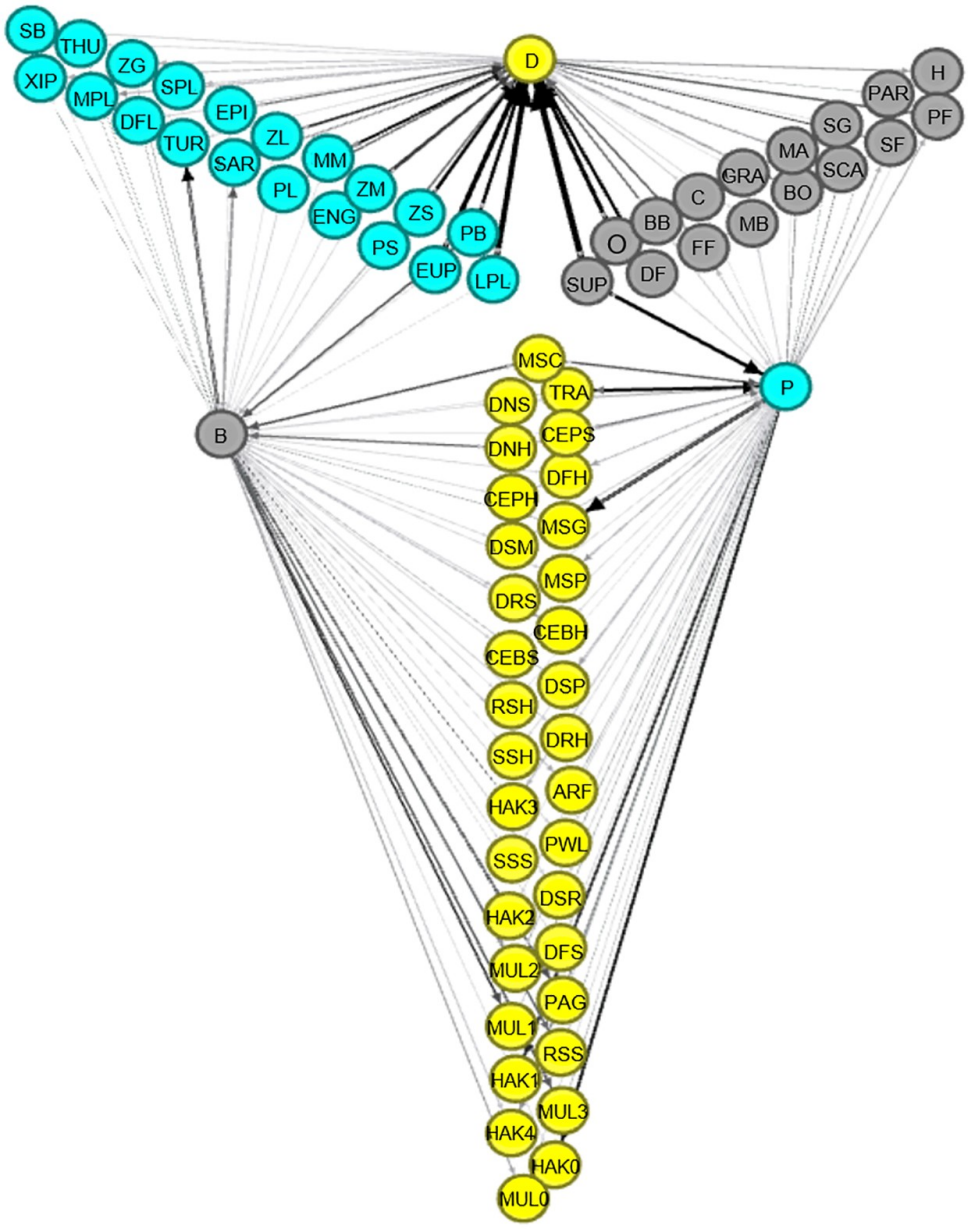
BPC explored by overall impact among 69 living functional groups. FGs are displaced on radial axes representing the domain which they belong to: pelagic (P, cyan), demersal (D, yellow), benthic (B, gray). Arrow thickness is proportional to the overall impact they have on the remaining domains as well as the impact FGs received by the domains (size nodes) they do not belong to. Higher overall impact (black arrow) are from the most impacting FGs to side nodes and from side nodes to the most impacted FGs detailed in Table 2.

The results of the MTI analysis are synthetized in Fig 6. High values of ɛ (represented by node size) resulted for groups in all three domains. In the benthic domain, groups with high ɛ_i_ were suprabenthos (SUP), macrobenthos carnivore (C), omnivore (O), scavenger (SCA) and detritus feeder (DF); in the demersal domain, high ɛ_i_ values were shown by demersal fish from slope (DFS), TRA, MSC, rays from shelf (RSH), sharks from slope (SSS) and decapods natant from the slope (DNS). Finally in the pelagic domain, large pelagic fish (LPL), euphausiids (EUP), European anchovy (ENG), zooplankton medium (ZM) and small (ZS) were the groups with highest ɛ_i_ (Fig 6). These groups exerted high overall cumulative impacts through a large set of small impacts (low values of MTI; see also S2 Fig), while medium pelagic fish (MPL), demersal piscivorous fish (DSP) and reptant decapods from shelf (DRH) were the groups with the highest single value of MTI. The main overall effect on BPC was exerted by a group of key actors as shown in Fig 7. In fact, considering both direct and indirect overall effects, SUP, LPL, EUP produced high impacts on groups pertaining to other domains, while mesopelagic fish jellyfish feeders (MSG), SAR and sea turtles (TUR) were primarily affected by couplers of benthic and pelagic domains (Fig 7). Generally, macro-benthic organisms had a positive impact on demersal (e.g. SUP on HAK) and pelagic groups (e.g. SUP on SAR). Overall, the main impacting FGs on the BPC spanned from benthic (e.g., SUP) to pelagic (e.g., LPL) and exerted their impact mainly through demersal FGs (Fig 7).

### BPC and mixed fisheries relationship

**Fig 8:**
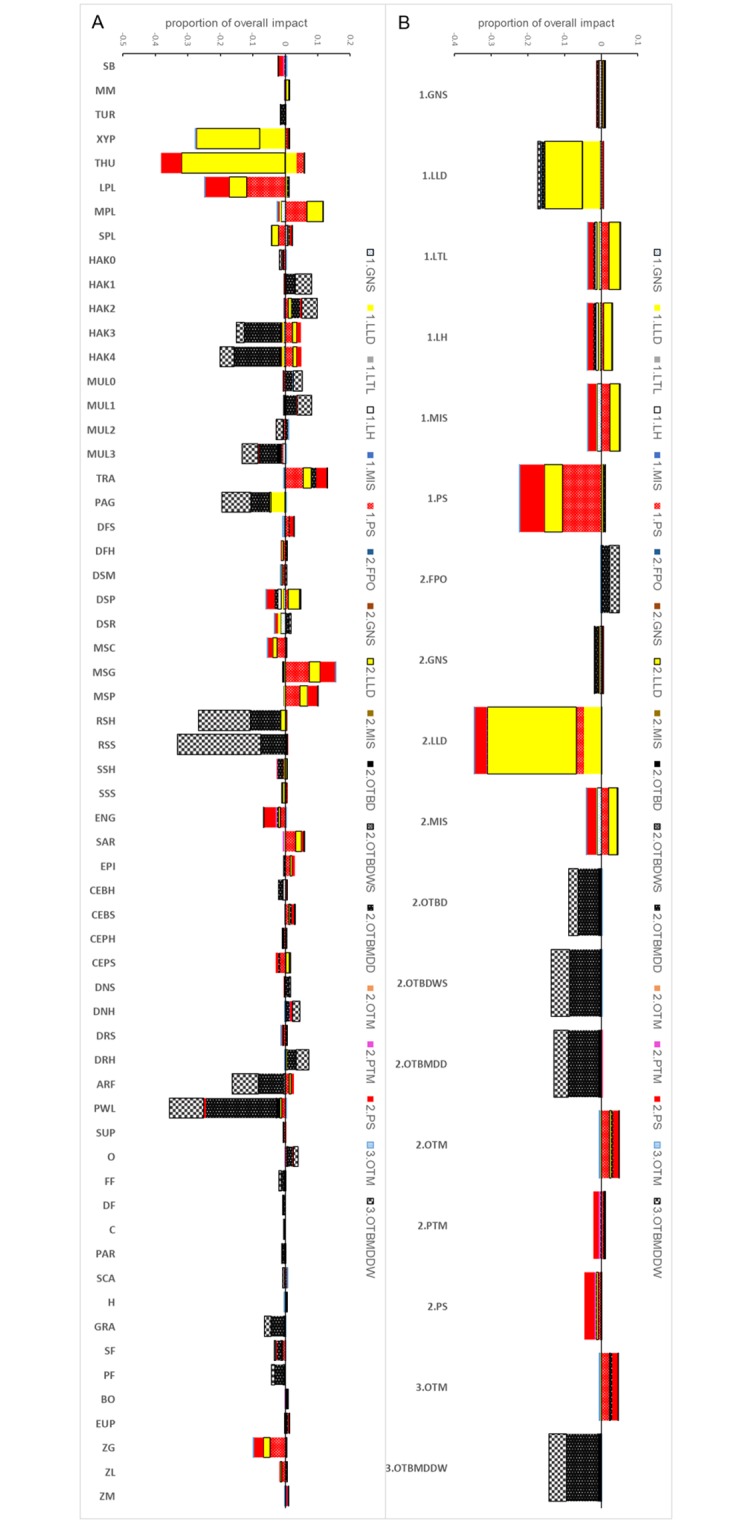
Results of the MTI analysis for fleets of the SoS ecosystem model. (A) overall impact across biological functional groups. (B) conflicts (negative values of MTI) and benefits (positive values of MTI) among fleet segments.

The MTI analysis showed fishing effects cascading on the food web (Fig 8A) as well as overall interactions among fleets (Fig 8B).

Bottom trawlers of LOA class 2 and 3 (2.OTB_MDD, 3.OTB_MDDW) had negative direct impact on main target demersal species such as large European hake (HAK3 and 4), red mullet (MUL3), rays and sharks (RSH and RSS), but had positive effects on hake and red mullet juvenile (HAK1 and 2; MUL0 and 1). Moreover, these fisheries had negative indirect impact on benthic organisms such as benthic decapods (mainly DNH, DRH) mediated by the depletion of their predators (Fig 8A). Similarly, longliners (1.LLD, 2.LLD) and purse-seiners (1.PS, 2.PS) had negative effects on their main pelagic targets, i.e., the large pelagic (XIP, THU, LPL) but also and indirect positive effect on several demersal species such as TRA, MSG, MSP and notably, HAK3 and HAK4 (Fig 8B). Interestingly purse-seiners have negative impact on jellyfish (ZG) which is not among their target species. HAK and TRA were bycatch species of pelagic pair trawlers (PTM) and midwater-mixed trawlers (OTM) and were indirectly favored by purse-seiners (LOA1 and 2) through the BPC (Fig 8A).

The MTI disentangled by fleet (Fig 8B) shows that, besides the strong intra-gear competition (negative values), bottom trawlers had a small, indirect and positive effect on pair and midwater-mixed trawlers. A large negative impact of 2.OTB_MDD on other bottom trawlers was evident as well as a positive effect on traps. A competition (negative impact) between LOA1 and LOA2 of both purse-seiners (GNS) and long-liners (LLD) appeared but purse-seiners had positive effects on 2-3.OTM (Fig 8B).

**Fig 9:**
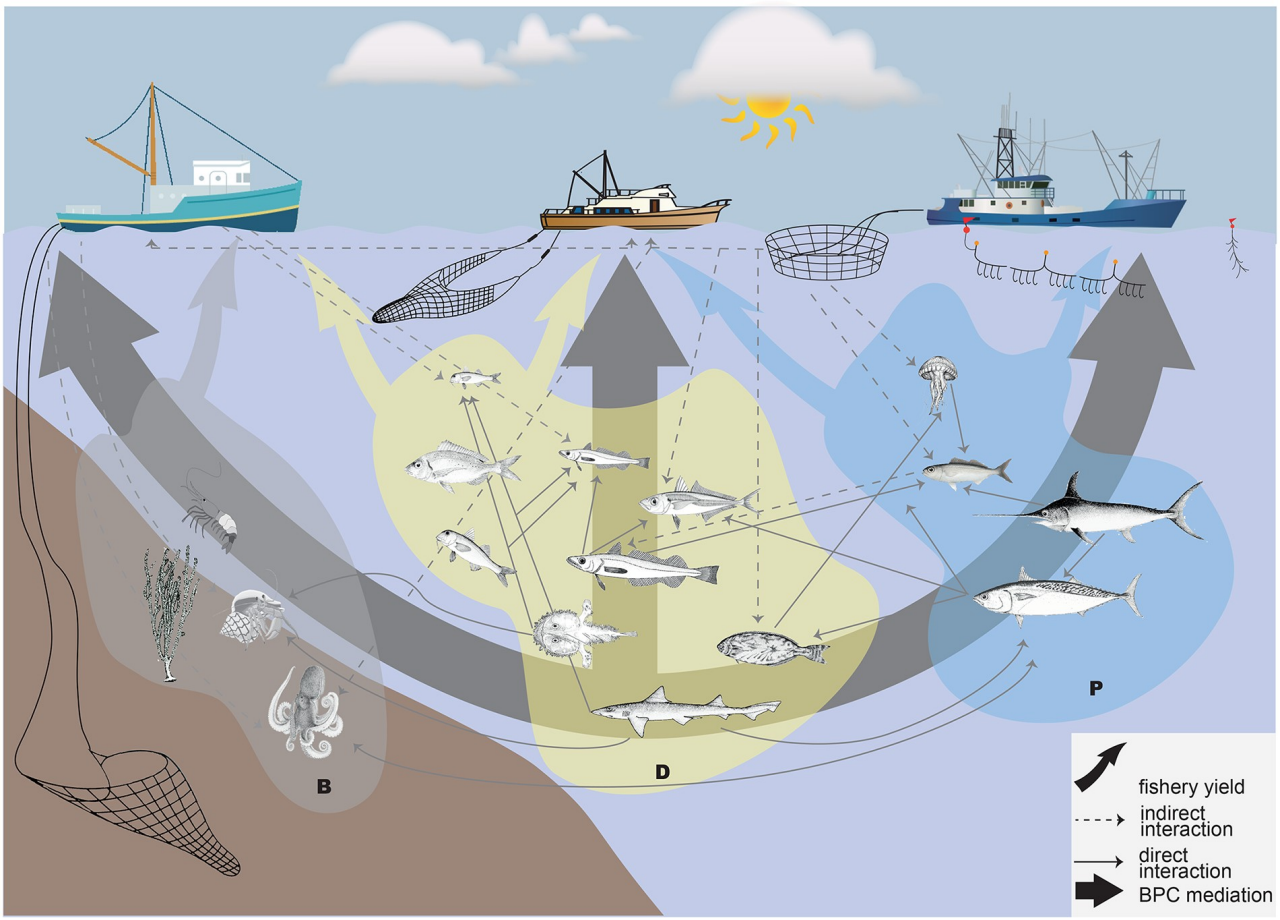
BPC mediates mixed fisheries: Main fleet and biological overall impacts (direct and indirect) through the three domains. Gray cloud = benthic domain, yellow cloud = demersal domain, blue cloud = pelagic domain. From left to right the fleets represent: bottom trawl, pair and mid-water trawl, purse seine and longline.

Fig 9 synthesizes the complex set of indirect interactions that connect bottom trawling to pelagic pair and midwater-mixed trawlers, showing that the positive effect of the bottom trawling on the latter is obtained promoting weak and diffused interactions that have a positive effect on TRA (e.g. sharks-large and medium pelagic fish-cephalopods-TRA). Similarly, the negative effect of purse seiners and long-liners on medium (MPL) and large pelagic fish (LPL), promotes an increase in jellyfish feeders such as EPI (mainly *Boops boops*) and MSG (mainly *Schedophilus medusophagus*) and a decrease in jellyfish (ZG). At the same time, biomass of predators such as benthic cephalopods from slope (CEBS), mesopelagic piscivorous fish (MSP) and DFS is released. However, forage fish and horse mackerel populations can increase so that predators such as European hake could benefit from this food resource (Fig 9).

## Discussion

### Model validation

The development of the SoS ecosystem model required reconciling data and parameters obtained from a variety of independent sources and for a very wide set of species. The efforts for producing an accurate ecosystem description started with the revision and comparison of different data sources but unavoidably encompassed adjustments with respect to data which were the smallest possible. The comparison between Ecopath inputs and parameters with those of stock assessments highlighted a good accuracy of the ecosystem model developed. The comparison also evidenced that, in order to have similar total mortalities in the two approaches, it is often resulting in higher natural mortality in Ecopath possibly for accommodating predation mortalities. Conversely fishing mortalities for target species resulted slightly smaller. The review of wide set of information regarding biological, domain and fisheries aspects for the development of SoS model highlighted local gaps (such as the information on the benthic infauna) and, general issues encountered when working in a context of multiple targets and mixed fisheries, such as the difficulties in defining a model area that overlap completely resources, exploitations and data available.

### Ecosystem structure and BPC

The food web of the Strait of Sicily described by our model resulted quite complex as suggested by the System Omnivory Index (SOI = 0.30), which is way above the average of 105 food web models [72] and other Mediterranean Ecopath models [68–70,75,76]. The SOI is considered a robust indicator in network analysis and its fluctuations should not be significantly influenced by the number of groups considered in the model [77]. Furthermore, Polis & Strong (1996) evidenced two main effects of omnivory: firstly, it diffuses the effects of productivity and consumption on the range of the trophic spectrum, secondly, consumers can increase by feeding on occasional prey, thus triggering a depression of their usual food sources. The first process could end up with a reinforced coupling across domains (i.e. benthic-pelagic) and the second could promote cascade effects.

The results have evidenced that most of the food web energetic fluxes were exchanged between bacteria and the various type of detritus (BD, SPOM, DC) and then, transferred from the detritus to the upper trophic levels. Bacteria and invertebrates of benthic and pelagic domains are characterized by high rate of production and consumption so that they handled the bulk of fluxes directly and indirectly linked to detritus. Thus, through a variety of feeding strategies, they were able to efficiently use detritus allowing an important coupling between planktonic and benthic invertebrates. Accordingly, other authors have suggested a prominent role of particulate organic matter, especially for marine snow (here SPOM) [9] across the whole vertical axis of the Mediterranean food web.

Moreveor, we have shown that high and medium trophic level items such as large pelagic fish (LPL), mesopelagic fish (MSC, MSP, MSG), *Trachurus* spp. (TRA), rays, sharks, cephalopods and demersal fish (DFH) had high ranks of overall effects. Medium pelagic (MPL), demersal piscivores (DSP) and reptant decapods from shelf (DRH) have high MTI values and fish that undergo through size/age related diet shifts (here red mullet and European hake) interact with a large number of prey and predators from all domains deeply contributing to BPC in space [78]. Most of these FGs indeed, are able to move on a large spatial scale and their life history is spent between shelf and slope domains where they play a key role in linking subsystems together (i.e. shelf-slope) at a wide scale [78,79] making ultimately the ecosystem more stable [78,80]. Moreover, most trophic interactions among components were low in terms of energetic flux but relevant for the number of links suggesting the existence of a large set of weak interactions. A primary importance has been given to this type of interactions in bio-diverse systems [79,81] because weak interactions serve to limit energy flow in a potentially strong consumer—resource interaction and, therefore, to inhibit over-consumption that destabilizes the dynamics of food webs [79,82].

### Role of ecosystem components on the BPC

The SoS has been an important fishing ground since ancient times and, at the same time it represents an ecosystem that largely contributes to Mediterranean biodiversity in terms of habitats and species [38]. Critically enough, however, several target species such as deep-water rose shrimp (PWL), hake (HAK) and swordfish (XIP) resulted overexploited and many non-commercial species suffer conspicuous fishing mortality mainly by large bottom trawlers, long-liners and purse-seiners. Furthermore, a high discard ratio (0.45), in particular benthic invertebrates and horse mackerel, resulted associated to bottom trawlers [63,83].

Our results show that fishery driven interactions are involved in BPC through their pervasive action on the whole food web and through indirect effects among fleets. Acting on resources linked by weak interactions, the fleets possibly promote cascade effects such as those shown here for the Strait of Sicily but possibly observable in other Mediterranean ecosystems.

The model proposed in this study does not include processes and effects linked to the habitat modification caused by bottom trawling as well as the effects of physical factors (i.e. temperature, marine currents, etc.) and the mass balance can facilitate the identification of main contributors, but their consistency may be strengthened in a next step by implementing the temporal dynamic approach i.e., the Ecosim module [84,85]. However, the MTI analysis is a proxy of a dynamic simulation [74] and some steps of the important interactions reported here (i.e. trawlers-mega_macrobenthos, epipelagic fish- jellyfish) have also been demonstrated by other authors in the Mediterranean [86–89]. Moreover, several benthic FGs such as suspension and filter feeders appeared negatively affected by trawling. These FGs are habitat formers and have been suggested to have a key role in BPC and in the carbon cycle [90], and are also vulnerable to fishing. In fact, filter feeders such as the deep sea corals *Isidella elongata* and *Funiculina quadrangularis* are critically endangered by trawlers in the SoS [91].

Benthic-pelagic coupling therefore appears to be shaped by multiple interactions among group of species (sp.-sp.), among fleets (fleet-fleet) as well as between fleet and group of species (fleet-sp.). Although the analysis of direct interactions suggests that main elements of BPC are mediated by wasp-waist groups (mainly mesopelagic fish crustacean feeders, MSC and epipelagic fish, EPI), which show great contribution to energy transfer as consumers and sources in the food web, the quantification of direct and indirect impacts highlighted the important role of other components in the BPC.

Interestingly, it has been recently suggested that the increasing depletion of top predators over the past decades, has led to a shift of fisheries towards forage fish and benthic invertebrates [92] that could have an important role in BPC [26]. Forage fish have been also recognized as wasp-waist species [93] exerting a double important role: top-down on their prey and bottom-up on their predators. The MTI and fluxes shown here suggest that many groups over wasp-waist species could play a role in the BPC regardless of which domain they belong to. Moreover, most of the target species are linked by weak interactions and the high erosion of biomass could make them highly sensitive to fishing and may lead to the rise of unexpected consumers [94] and trophic cascades into a scenario of simplified and degraded ecosystems.

Moreover, the result of this study shows that BPC mediates the Mediterranean mixed fishery through an intricate combination of biological and fisheries interactions and therefore the idea of unrelated demersal and pelagic fisheries is to be set aside.

Overall, this study provides a novel and comprehensive quantitative integration of a large amount of information on biomass density, population dynamics and trophic interactions in the Strait of Sicily, contributing to a deeper understanding of the functioning of the whole food web, and offers a novel assessment of the overall effects of fishing in an ecological Mediterranean framework.

## Supporting information

S1 Appendix Modelling approach, balancing strategy and pedigree. (DOCX)

S1 Table Sources and references used for each functional group representing the Strait of Sicily food web model. Biomass (*B*); diet information used to build the diet matrix; production per unit of biomass (*P*/*B*); consumption per unit of biomass (*Q*/*B*). F = fishing mortality, M = natural mortality, Z = total mortality.
(DOCX)

S2 Table Input parameters and main outputs (in bold) of the food web model. For each functional group (FG) are detailed inputs: biomass (B; t km^−2^), production/biomass ratio (P/B; yr^−1^), consumption/biomass ratio (Q/B; yr^−1^); Landing and Discards are expressed in t km^−2^ year^−1^. Outputs: trophic level (TL), ecotrophic efficiency (EE; for most of the FG except for BO, EUP, MB), production/consumption (P/Q), respiration/assimilation (R/A), omnivory index (OI). Dom = Domain: p = pelagic, d = demersal, b = benthic.
(DOCX)

S3 Table Matrix of diet used as input. Raw data across functional groups of predators in columns and preys in rows.
(DOCX)

S4 Table Ecological and fishery related indices across modelled Mediterranean ecosystems: Gulf of Gabes (Hattab et al. 2013), Catalan Sea (Coll et al. 2009_early 2000s), Adriatic Sea (Coll et al. 2007), Aegean Sea (Tsagarakis et al. 2010), Greek Ionian Sea (Moutopoulos et al. 2013), Mediterranean Sea (Piroddi et al. 2015_global). B total biomass (exluding detritus), Q total consumption, Ex sum of all exports, R sum of all respiratory flow, FD sum of all flows into detritus, P sum of all productions, TST total system throughput, PP net primary production, TE mean transfer efficiency, C capacity, O overhead, A ascendency, FCI model stress, Y catches, TLm mean trophic level of catches, Y/PP gross efficiency.
(DOCX)

S1 Fig Summary of the diet matrix of the food web model. Proportion of preys (rows) in the diet of the predators (columns) in ranks. Fraction of import in the diet (fraction of energy assumed to be taken out of the system) are also indicated.
(TIF)

S2 Fig Mixed trophic impact matrix. Rows are impacting FGs and columns impacted FGs.
(TIF)
