# Supplementary material for: Benthic-pelagic coupling mediates interactions in Mediterranean mixed fisheries: An ecosystem modeling approach
Source: PLoS One. 2019 Jan 15;14(1):e0210659. doi: 10.1371/journal.pone.0210659 (PMC6333361; doi:10.1371/journal.pone.0210659)
Supplement: S1 Appendix — (DOCX) [file pone.0210659.s001.docx]

**S1 Appendix**

**Modelling approach, balancing strategy and pedigree**

Ecopath is based on the balance of energy flows and biomass of functional groups, which represents species or groups of species having the same ecological and trophic function. Moreover, it enables to account for the effect of fishing activities on each functional group. The energy balance within and among each group is ensured through two linear equations: one that sets the production of each functional group (Pi) equal to predator consumption (M2i), export from the system (e.g., fisheries yield, Yi), other mortality (M0i=1-Ecotrophic efficiency, EE) and biomass accumulation (BAi) and another that sets food consumption (Qi) equal to production (Pi), respiration (Ri), and unassimilated food of each group (UNi). The input parameters for each functional group are biomass (Bi), production rate (P/Bi), which is equal to total mortality rate [1], consumption rate (Q/Bi), diet (diet matrix DCij as a fraction of prey i in the diet of predator j), exports EXi by different fishing activities including discards, and the unassimilated food ratio (UNi) for each group. The growth efficiency (P/Q), the respiration rate (R/B) and the fraction of the production that is either utilized within the system by predators or exported (termed Ecotrophic Efficiency, EE) by group are usually estimated by the model, allowing assessment of whether the food web model is balanced, namely when P/Q<0.5 for all groups, R/B is consistent with the metabolism of the group and EE<1 for all groups; i.e., high values for top predators and small organisms [2].

The initial Ecopath model based on input parameters as described in S1 table was evaluated though a pre-balancing analysis (PREBAL [3,4]) to assess whether data were coherent with some basic ecological rules. A number of diagnostics were used in this study in order to test: biomass across taxa/trophic levels (where biomass should span 5–7 orders of magnitude and slope on log scale should be 5–10% decline), vital rates across taxa/trophic levels (should be a general decline with increasing trophic level), the P/Q rate (should be <0.5), the Respiration/Assimilation rate (must be <1) and the Ecotrophic Efficiency (EE) (must be <1) (S2 Table).

Groups of invertebrates (macro-benthos, cephalopods and decapods both from shelf and slope), demersal fish from shelf (DFH) and swordfish generated an initial EE value higher than 1, revealing that these groups were over-predated/caught. To reduce the EE of these groups, we corrected our diet matrix inputs, especially for groups for which data were not originally from the modeled area. Diet composition modifications were preferred to other parameter changes such as biomass or production rate, particularly for groups whom biomass data and growth parameter existed. The high EE in swordfish was caused by high catches instead of natural predation, hence we introduced an immigration rate (0.025 tonnes km^-2^ y^-1^) in order to balance catches.

The pedigree routine available in EwE was used in order to evaluate data quality by categorizing the different input sources. Resulted a pedigree of 0.74 suggesting a good quality of data used for the construction of the model (S2 Table, S3 Table). Moreover, ecosystem indicators were calculated with the Network analysis routine embedded in EwE. These indicators include Transfer Efficiency (TE) and proportion of flows type that were compared with other Mediterranean ecosystem models (S4 Table) in order to diagnose for possible flaws (e.g., [5–7]).

**References S1 appendix**

1. Pauly D, Christensen V, Walters C. Ecopath, Ecosim, and Ecospace as tools for evaluating ecosystem impact of fisheries. ICES J Mar Sci. 2000;57: 697–706. doi:10.1006/jmsc.2000.0726

2. Christensen V, Walters CJ. Ecopath with Ecosim: Methods, capabilities and limitations. Ecological Modelling. 2004. pp. 109–139. doi:10.1016/j.ecolmodel.2003.09.003

3. Link JS. Adding rigor to ecological network models by evaluating a set of pre-balance diagnostics: A plea for PREBAL. Ecol Modell. 2010;221: 1580–1591. doi:10.1016/j.ecolmodel.2010.03.012

4. Heymans JJ, Coll M, Link JS, Mackinson S, Steenbeek J, Walters C, et al. Best practice in Ecopath with Ecosim food-web models for ecosystem-based management. Ecol Modell. 2016;331: 173–184. doi:10.1016/j.ecolmodel.2015.12.007

5. Heymans JJ, Coll M, Libralato S, Morissette L, Christensen V. Global Patterns in Ecological Indicators of Marine Food Webs: A Modelling Approach. PLoS One. 2014;9: e95845. doi:10.1371/journal.pone.0095845

6. Heymans JJ, Coll M, Libralato S, Christensen V. Ecopath Theory, Modeling, and Application to Coastal Ecosystems. Treatise Estuar Coast Sci. 2011;9: 93–113. doi:10.1016/B978-0-12-374711-2.00905-0

7. Libralato S, Solidoro C. Bridging biogeochemical and food web models for an End-to-End representation of marine ecosystem dynamics: The Venice lagoon case study. Ecol Modell. 2009;220: 2960–2971. doi:10.1016/j.ecolmodel.2009.08.017
