# Supplementary material for: Benthic-pelagic coupling mediates interactions in Mediterranean mixed fisheries: An ecosystem modeling approach
Source: PLoS One. 2019 Jan 15;14(1):e0210659. doi: 10.1371/journal.pone.0210659 (PMC6333361; doi:10.1371/journal.pone.0210659)
Supplement: S1 Table — Biomass (B); diet information used to build the diet matrix; production per unit of biomass (P/B); consumption per unit of biomass (Q/B). F = fishing mortality, M = natural mortality, Z = total mortality. (DOCX) [file pone.0210659.s002.docx]

**S1 Table.**

| **N. Functional group (code)** | **(number of taxa)**  ***main Taxa*** | **Biomass** | **Diet** | **P/B** | **Q/B** |
| --- | --- | --- | --- | --- | --- |
| **1.Sea birds (SB)** | \| (19) \| \| --- \| \| *Hydrobathes pelagicus* \| \| *Larus melanocephalus* \| \| *Larus fuscus* \| \| *Phalacrocorax carbo*  *Sterna spp.* \| | Abundance data from South of Sicily (ISPRA report) has been transformed to t·km−2 with the mean body weight per species (Del Hoyo et al. 1992) | Albores-Barajas 2009,  Fasola et al.1989,  Sarà 1993,  UNEP_RAC/SPA report 2014 | Piroddi et al. 2015,  Ristow et al.1990 | Nagy 1987  Piroddi et al. 2015 |
| **2.Marine mammals (MM)** | (8)  *Balenoptera physalus*  *Delphinus delphis*  *Globicephala melas*  *Grampus griseus*  *Physeter macrocephalus*  *Stenella coeruleoalba*  *Tursiops truncatus*  *Ziphius cavirostris* | Notarbartolo di Sciara et al. 2003, report MSFD initial assessment Malta. Status and conservation of cetaceans in the Sicily Channel/Tunisian plateau RAC/SPA MedOpenSeas Project 2014, Units of individuals·km−2 has been transformed to t·km−2 with the mean body weight per species (Carwardine & Camm, 1998 and www.fishbase.org) | Santos et al. 2001, Blanco et al. 2001, Canese et al. 2006  Reeves & Notarbartolo di Sciara 2006, Cotté et al. 2009, Bearzi et al.  2011, Pauly et al. 1998 | Coll et al. 2006, 2008, 2009,  Piroddi et al. 2015,  Trites & Pauly 1998 | Coll et al. 2006, 2008, 2009  Innes et al.1987 |
| **3.Sea turtles (TUR)** | (3)  *Caretta caretta* | Average based on values reported by Coll et al. 2006, 2008, 2009,  Piroddi et al. 2015 | Birdlife www.birdlife.org; Piroddi et al. 2010,  Piroddi et al. 2011,  Moutopoulos et al. 2013, Russo et al. 1999 | Casale et al. 2009, Coll et al. 2006, 2008, 2009,  Piroddi et al. 2015 | Coll et al. 2006, 2008, 2009,  Piroddi et al. 2015 |
| **4.Sword fish (XIP)** | (1)  *Xiphias gladius* | Fishstat www.fao.org; http://www.iccat.int/en/assess.htm | Bello 1991  Stergiou and Karpouzi 2001 | http://www.iccat.int/en/assess.htm,  Piroddi et al. 2015 | Estimate based on values reported for large pelagics by Hattab et al. 2013, Moutopoulos et al. 2013 |
| **5.Bluefin tuna (THU)** | (2)  *Thunnus thynnus*  *Thunnus alalunga* | Fishstat www.fao.org; http://www.iccat.int/en/assess.htm | Stergiou and Karpouzi 2001, Sarà et al.2007; Sinopoli et al. 2004 | http://www.iccat.int/en/assess.htm Piroddi et al. 2015 | Estimate based on values reported for large pelagics by Hattab et al. 2013, Moutopoulos et al. 2013 |
| **6.Large pelagic fish (LPL)** | (4)  *Coriphaena hyppurus*  *Euthynnus alletteratus* | Fishstat www.fao.org; http://www.iccat.int/en/assess.htm Piroddi et al. 2015 | Coll et al. 2007, Massutì et al. 1998, Stergiou and Karpouzi 2001 | http://www.iccat.int/en/assess.htm,  Piroddi et al. 2015 | Hattab et al. 2013, Moutopoulos et al. 2013 |
| **7.Medium pelagic fish (MPL)** | (13)  *Auxis rochei*  *Sarda sarda*  *Scomber spp.*  *Sphyraena sphyraena* | Trawl survey (MEDITS)  Sicily straits  2004-2005 | Akadje et al. 2013,  Hattour 2000 | *Z* = *F* + *M*; M = empirical  equation from Pauly  (1980) | www.fishbase.org |
| **8.Other small pelagic fish (SPL)** | (9)  *Sardinella aurita*  *Spicara spp.*  *Sprattus sprattus* | Trawl survey (MEDITS)  Sicily straits  2004-2005 | Lomiri 2008  Morote et al 2008  Pinnegar & Polunin 2000 | https://stecf.jrc.ec.europa.eu/reports/medbs,  Brando et al. 2004 | www.fishbase.org |
| **9-13.European hake (HAK)** | *Merluccius merluccius*  HAK0(<6cm)  KAH1(6.1-12cm)  HAK2(12.1-22cm)  HAK3(22.1-41cm)  HAK4(>41.1cm) | Trawl survey (MEDITS)  Sicily straits  2004-2005 and  https://stecf.jrc.ec.europa.eu/reports/medbs | Sinopoli et al. 2012 | https://stecf.jrc.ec.europa.eu/reports/medbs and local data (IAMC-CNR Mazara del Vallo) | www.fishbase.org |
| **14-17.Red mullet (MUL)** | *Mullus barbatus*  MUL0(<8cm)  MUL1(8.1-12cm)  MUL2(12.1-17cm)  MUL3(>17cm) | Trawl survey (MEDITS)  Sicily straits  2004-2005 and  https://stecf.jrc.ec.europa.eu/reports/medbs | Cherif et al. 2011, Lipari et al. 1998 | https://stecf.jrc.ec.europa.eu/reports/medbs and local data (IAMC-CNR, Castellammare del Golfo) | Carpentieri 2007 |
| **18.Horse mackerel (TRA)** | (3)  *Trachurus trachurus*  *Trachurus mediterraneus* | Trawl survey (MEDITS)  Sicily straits  2004-2005 | Ben Salem 1988, Jardas et al 2004  Rumolo et al. 2017 Santic et al 2004 | Pauly (1980) | www.fishbase.org |
| **19.Pandora (PAG)** | (1)  *Pagellus erythrinus* | Trawl survey (MEDITS)  Sicily straits  2004-2005 | Fanelli et al. 2011 | Pauly (1980), https://stecf.jrc.ec.europa.eu/reports/medbs | www.fishbase.org |
| **20.Demersal fish (slope) (DFS)** | (27)  *Lophius budegassa*  *Lophius pisctorius* | Trawl survey (MEDITS)  Sicily straits  2004-2005 | Stagioni et al. 2013, Stergiou and Karpouzi 2002 | Coll et al. 2007 | Madurell and Cartes 2005,  www.fishbase.org |
| **21.Demersal fish crustacean feeders (shelf) (DFH)** | (57)  *Aphia minuta*  *Argentina sphyraena*  *Arnoglossus laterna*  *Citharus linguatula*  *Chelidonichthys lucerna*  *Lithognathus mormyrus*  *Pagellus acarne*  *Trisopterus capelanus* | Trawl survey (MEDITS)  Sicily straits  2004-2005 | Boudaya et al. 2007, 2008, Carpentieri et al. 2007, Fanelli et al 2011, Fanelli et al. 2009a, Filiz & Toğulga 2009, Menif 2000 | Colloca et al. 1997,  Mertz and Myers 1998, www.fishbase.org | Carpentieri 2007,  www.fishbase.org |
| **22.Demersal fish mixed food (shelf) (DSM)** | (32)  *Mullus surmuletus*  *Serranidae*  *Solea solea* | Trawl survey (MEDITS)  Sicily straits  2004-2005 | Arculeo et al.1993, Molinero and Flos1991, Ouannes-Ghorbel et al. 2005, Ktari 1979,  Pipitone MSc thesis 1987 | Arreguìn-Sanchèz et al. 2002,  Beverton & Holt 1957,  Pauly (1980) | Carpentieri 2007,  www.fishbase.org |
| **23.Demersal fish piscivorous (shelf) (DSP)** | (15)  *Conger conger*  *Seriola dumerili*  *Trachinidae.*  *Zeus faber* | Trawl survey (MEDITS)  Sicily straits (GSA15-16)  2004-2005 | Anastasopoulou et al 2013, Andaloro & Pipitone 1995, Esposito et al. 2009, Ismen et al. 2013, Morte et al 1999 | Dìaz Lòpez et al. 2008 | www.fishbase.org |
| **24.Demersal fish rocky (shelf) (DSR)** | (42)  *Dentex dentex*  *Diplodus spp.*  *Epinephelus marginatus*  *Oblada melanura*  *Sarpa salpa*  *Scorpaena spp.*  *Sparus aurata*  *Spondyliosoma cantharus* | Trawl survey (MEDITS)  Sicily straits  2004-2005 | Abdelkader and Ktari 1986, Badalamenti et al. 1993, Bradai et al. 1998a, Bradai and Bouain 1990, Carasson and Matallanas 1998, Fabi et al 1998, Kitsos et al. 2008, Morte et al. 2001, Pepe et al. 1996,1998 | Mertz and Myers 1998,  www.fishbase.org | www.fishbase.org |
| **25.Mesopelagic fish crustacean feeders(slope) (MSC)** | (65)  *Capros aper,*  *Coelorinchus caelorhincus; Epigonus denticulatus, Hoplostethus mediterraneus, Lampanyctus crocodilus, Lepidopus caudatus, Macroramphosus scolopax*  *Myctophidae*  *Nezumia aequalis,*  *Nettastoma melanurum* | Trawl survey (MEDITS)  Sicily straits  2004-2005 | Carpentieri et al 2016, Fanelli and Cartes 2010, Macpherson 1979, Matallanas 1982, Stefanescu and Cartes 1992, Server et al 2008 | Arreguìn-Sànchez et al. 2002, Heymans 2005, Tsarin, 1994 | www.fishbase.org |
| **26.Mesopelagic fish jellyfish feeders(slope) (MSG)** | (3)  *Centrolophus niger*  *Cubiceps gracilis*  *Schedophilus medusophagus,* | Trawl survey (MEDITS)  Sicily straits  2004-2005 | Battaglia et al 2014 | Pauly (1980) | www.fishbase.org |
| **27.Mesopelagic fish piscivorous (slope) (MSP)** | (3)  *Chauliodus sloani*  *Ruvettus pretiosus*  *Stomias boa boa* | Trawl survey (MEDITS)  Sicily straits  2004-2005 | Butler et al. 2001, Vasilakopoulos et al. 2011 | Guènette and Morato 2001,  www.fishbase.org | www.fishbase.org |
| **28.Rays and skates (shelf) (RSH)** | (17)  *Dasiatys pastinaca*  *Raja spp.* | Trawl survey (MEDITS)  Sicily straits  2004-2005 | Bradai et al 2012,  Cannizzaro et al. 1995,  Capapé 1976, 1977, Capapé and Azouzi 1976, Follesa et al 2010, Navarro et al 2013, Kadri et al. 2013, Vannucci et al. 2006 | Pauly 1980 | www.fishbase.org |
| **29.Rays and skates (slope) (RSS)** | (4)  *Dipturus oxyrinchus*  *Leucoraja circularis* | Trawl survey (MEDITS)  Sicily straits  2004-2005 | Ebert and Bizzarro 2007,  Mulas et al. 2015,  Kadri et al. 2013 | Pauly 1980 | www.fishbase.org |
| **30.Sharks (shelf) (SSH)** | (9)  *Mustelus spp.*  *Scyliorhinus spp.* | Trawl survey (MEDITS)  Sicily straits  2004-2005 | Stergiou and Carpouzi 2002, Bradai et al.2012,  Martinho et al. 2012 | Pauly 1980,  https://stecf.jrc.ec.europa.eu/reports/medbs | www.fishbase.org |
| **31.Sharks (slope) (SSS)** | (12)  *Galeus melastomus*  *Oxynotus centrina*  *Squalus blainville* | Trawl survey (MEDITS)  Sicily straits  2004-2005 | Bradai et al.2012,  Capapé 1975, Fanelli et al. 2009c, Martinho et al. 2012, Stergiou and Karpouzi 2002, Valls et al. 2011 | Pauly (1980) | www.fishbase.org |
| **32.European anchovy (ENG)** | (1)  *Engraulis encrasicolus* | Trawl survey (MEDITS)  Sicily straits  2004-2005,  https://stecf.jrc.ec.europa.eu/reports/medbs, Echosurvey AMECO 2006 | Catalán et al. 2010,  Rumolo et al. 2016 | https://stecf.jrc.ec.europa.eu/reports/medbs | Plounevez and Champalbert, 2000,  Tudela and Palomera 1995 |
| **33.European pilchardus (SAR)** | (1)  *Sardina pilchardus* | Trawl survey (MEDITS)  Sicily straits  2004-2005,  https://stecf.jrc.ec.europa.eu/reports/medbs, Echosurvey AMECO 2006 | Costalago and Palomera 2014, Rumolo et al. 2016, Server et al. 2005 | https://stecf.jrc.ec.europa.eu/reports/medbs | www.fishbase.org |
| **34.Epipelagic fish (EPI)** | (8)  *Boops boops*  *Chromis chromis* | Trawl survey (MEDITS)  Sicily straits  2004-2005 | Derbal and Kara 2008, Moreno and Castro 1995, Milisenda et al. 2014 | Brando et al. 2004 | www.fishbase.org |
| **35.Cephalopods benthic (shelf) (CEBH)** | (16)  *Eledone spp.*  *Octopus vulgaris*  *Sepia spp.* | Trawl survey (MEDITS)  Sicily straits  2004-2005 | Guerra 1978, Najai and Ktari 1979, Šifner and Vrgoč 2009, Zghidi et al. 2003 | Empirical equation (Bray 2001) | Boyle 1990, Wells and Clarke 1996 |
| **36.Cephalopods benthic (slope) (CEBS)** | (8)  *Neorossia caroli*  *Octopus salutii*  *Scaeurgus unicirrhus* | Trawl survey (MEDITS)  Sicily straits  2004-2005 | Bello 1991,  Quetglas et al. 2001 | Empirical equation (Bray 2001) | Boyle 1990, Wells and Clarke 1996,  Empirical equation (Cammen 1980) |
| **37.Cephalopods pelagic (shelf) (CEPH)** | (5)  *Alloteuthis spp.*  *Loligo vulgaris* | Trawl survey (MEDITS)  Sicily straits  2004-2005 | Pierce et al. 1994,  Valls et al. 2015 | Empirical equation (Bray 2001) | Boyle 1990, Wells and Clarke 1996,  Empirical equation (Cammen 1980) |
| **38.Cephalopods pelagic (slope) (CEPS)** | (25)  *Illex coindetii*  *Todarodes sagittatus*  *Todaropsis eblanae* | Trawl survey (MEDITS)  Sicily straits  2004-2005 | Marabello 1996,  Lelli et al. 2005,  Rosas-Luis et al. 2014 | Empirical equation (Bray 2001) | Boyle 1990, Wells and Clarke 1996,  Empirical equation (Cammen 1980) |
| **39.Decapods natant (slope) (DNS)** | (38)  *Aristeus antennatus*  *Pasiphaea spp.*  *Plesionika heterocarpus*  *Polycheles typhlops* | Trawl survey (MEDITS)  Sicily straits  2004-2005 | Cartes 1994, 1995 | https://stecf.jrc.ec.europa.eu/reports/medbs,  Empirical equation (Bray 2001) | Maynou and Cartes 1998,  Empirical equation (Cammen 1980) |
| **40.Decapods natant (shelf) (DNH)** | (23)  *Aegaeon spp.*  *Solenocera membranacea* | Trawl survey (MEDITS)  Sicily straits  2004-2005 | Cartes 1994, 1995 | Empirical equation (Bray 2001) | Maynou & Cartes, 1998  Empirical equation (Cammen 1980) |
| **41.Decapods reptant (slope) (DRS)** | (27)  *Macropipus tuberculatus*  *Munida iris*  *Nephrops norvegicus*  *Paromola cuvieri* | Trawl survey (MEDITS)  Sicily straits  2004-2005 | Cartes 1993,  Cristo 1998 | https://stecf.jrc.ec.europa.eu/reports/medbs,  Empirical equation (Bray 2001) | Maynou and Cartes 1998,  Empirical equation (Cammen 1980) |
| **42.Decapods reptant (shelf) (DRH)** | (41)  *Homarus gammarus*  *Liocarcinus spp.*  *Maja squinado*  *Palinurus elephas*  *Scyllarides latus*  *Squilla mantis* | Trawl survey (MEDITS)  Sicily straits  2004-2005 | Abello’ and Cartes 1987, Froglia and Giannini 1989, Goñi et al. 2001 | https://stecf.jrc.ec.europa.eu/reports/medbs,  Empirical equation (Bray 2001) | Maynou and Cartes 1998,  Empirical equation (Cammen 1980) |
| **43.Giant red shrimp (ARF)** | (1)  *Aristaeomorpha foliacea* | Trawl survey (MEDITS)  Sicily straits  2004-2005 | Gristina et al. 1992 | https://stecf.jrc.ec.europa.eu/reports/medbs | Maynou & Cartes, 1998  Empirical equation (Cammen 1980) |
| **44.Deep water rose shrimp (PWL)** | (1)  *Parapeenaeus longirostris* | Trawl survey (MEDITS)  Sicily straits  2004-2005 | Cartes 1995,  Kapiris, 2004 | https://stecf.jrc.ec.europa.eu/reports/medbs | Empirical equation (Cammen 1980) |
| **45.Suprabenthos (SUP)** | (107)  *Alpheus glaber*  *Amphipoda*  *Cumacea*  *Isopoda*  *Mysidacea* | Trawl survey (MEDITS)  Sicily straits  2004-2005 | Fanelli et al. 2009b, Polunin et al. 2001 | Cartes et al. 2011,  Romano et al. 2016,  Empirical equation (Bray 2001) | Madurell and Cartes 2005  Empirical equation (Cammen 1980)  Empirical equation (Brey 2010) |
| **46-55.Macrobenthos** | **(C)** Carnivores (84)  **(DF)** Detritus-feeders (100)  **(FF)** Filter feeders (169)  **(G)** Grazers (5)  **(H)** Herbivores (13)  **(O)** Omnivores (112)  **(PAR)** Parasites (10)  **(PF)** Particulate feeders (6)  **(SCA)** Scavengers (9)  **(SF)** Suspension feeders (9) | Trawl survey (MEDITS)  Sicily straits  2004-2005 implemented by experimental sampling in North-West Sicily (Romano et al. 2016) | Agnetta et al. 2013, Berthon 1987, Coma et al. 1995, Fauchald & Jumar 1979,  Frantzis et al. 1988, Greze 1968, Opitz 1996, Pearson and Gage 1984, Ribes et al. 1999 | Romano et al. 2016 | Empirical equation (Brey 2010) |
| **56.Meiobenthos (BO)** | _ | Ecopath estimate (0.95 EE) | General knowledge | Romano et al. 2016 | Empirical equation (Brey 2010) |
| **57.Euphausiids (EUP)** | (9)  *Meganyctiphanes norvegica*  *Nyctiphanes couchii* | Ecopath estimate (0.95 EE) | General knowledge | Lindley, 1982  Mackinson and Daskalov 2007 | Mackinson and Daskalov 2007 |
| **58. Zooplankton jelly-fish (ZG)** | (10)  *Aurelia aurita*  *Pelagia noctiluca*  *Rhizostoma pulmo*  *Siphonophorae*  *Thaliacea* | guesstimate from Aissi et al. 2014 | Canepa et al. 2014,  Sabatés et al. 2010,  Tecchio et al. 2013 | Coll et al. 2006,  Tecchio et al. 2013 | Coll et al. 2006,  Tecchio et al. 2013 |
| **59-61.Zooplankton** | **(ZS)** Small  **(ZM)** Medium  **(ZL)** Large | COPERNICUS MedMFC OPATM-BFM | adapted from OPATM-BFM results | OPATM-BFM | OPATM-BFM |
| **62.Pelagic bacteria (PB)** | _ | COPERNICUS MedMFC OPATM-BFM | suspended detritus | OPATM-BFM | OPATM-BFM |
| **63.Benthic bacteria (BB)** | _ | guesstimate from Danovaro et al. 2015, Mirto et al. 2004, Šestanovič et al. 2005 | benthic detritus | OPATM-BFM | OPATM-BFM |
| **64-66.Phytoplankton producer** | **(PS)** phytoplankton small  **(PL)** phytoplankton large  **(DFL)** dinoflagellates | COPERNICUS MedMFC OPATM-BFM | _ | OPATM-BFM | _ |
| **67.Microphytobenthos (MB)** | _ | Ecopath estimate (0.90 EE) | _ | Duarte and Cebrián 1996 | _ |
| **68.Seagrass (SG)** | *Posidonia oceanica* | Estimate from Di Carlo et al. 2007 | _ | Badalamenti et al. 2006,  Duarte and Chiscano 1999 | _ |
| **69.Macroalgae (MA)** | *Dyctiotales*  *Cystoseira spp.* | Estimate from experimental sampling North-West Sicily (unpublished authors’data) | _ | Duarte and Cebrián 1996 | _ |
| **70.Detritus Carrion (DC)** | _ | Estimate from discards (see M&M) | _ | _ | _ |
| **71.Suspended Particulate Organic Matter (SPOM)** | _ | COPERNICUS MedMFC OPATM-BFM | _ | _ | _ |
| **72.Benthic Detritus (BD)** | _ | COPERNICUS MedMFC OPATM-BFM | _ | _ | _ |

**References S1 table**

Abdelkader, B. and Ktari, M.. (1986) Régime alimentaire des Dentés (genre Dentex), Poissons, Sparidés de Tunisie. Bull. Soc. Sc. Nat. Tunisie 17, 19–25.

Abelló, P. and Cartes, J. (1987) Observations on the diet of Liocarcinus depurator (L.) (Brachyura: Portunidae) in the Catalan Sea. agris.fao.org.

Agnetta, D., Bonaviri, C., Badalamenti, F., Scianna, C., Vizzini, S. and Gianguzza, P. (2013) Functional traits of two co-occurring sea urchins across a barren/forest patch system. Journal of Sea Research 76, 170–177.

Akadje, C., Diaby, M., Le Loc’h, F., Konan, J.K. and N’Da, K. (2013) Diet of the barracuda Sphyraena guachancho in Côte d ’ Ivoire ( Equatorial Eastern Atlantic Ocean ). Cybium 37, 285–293.

Albores-Barajas, Y.V., Massa, B. and Soladatini, C. (2009) Ecologia riproduttiva dell’uccello delle tempeste mediterraneo (Hydrobates pelagicus melitensis). Alula 1, 159–162.

Anastasopoulou, A., Mytilineou, C.H., Lefkaditou, E., et al. (2013) The diet and feeding ecology of Conger conger (L. 1758) in the deep waters of the Eastern Ionian Sea. Mediterranean Marine Science 14, 365–368.

Andaloro, F. and Pipitone, C. (1997) Food and feeding habits of the amberjack, Seriola dumerili in the Central Mediterranean Sea during the spawning season. Cahiers de Biologie Marine 38, 91–96.

Arculeo, M., Froglia, C. and Riggio, S. (1993) Food partitioning between Serranus scriba and Scorpaena porcus (Perciformes) on the infralittoral ground of the South Tyrrhenian Sea. Cybium 17, 251–258.

Badalamenti, F., Di Carlo, G., D’Anna, G., Gristina, M. and Toccaceli, M. (2006) Effects of dredging activities on population dynamics of Posidonia oceanica (L.) Delile in the Mediterranean sea: the case study of Capo Feto (SW Sicily, Italy). Hydrobiologia 555, 253–261.

Badalamenti, F., D’Anna, G., Fazio, G., Gristina, M. and Lipari, R. (1993) Relazioni trofiche tra quattro specie ittiche catturate su differenti substrati nel Golfo di Castellammare (Sicilia N/O). Biologia Marina Mediterranea 1, 145–150.

Battaglia, P., Musolino, S., Esposito, V., Ammendolia, G., Consoli, P., Andaloro, F. and Romeo, T. (2014) Feeding habits of juvenile fishes belonging to three medusivorous species (Centrolophidae and Nomeidae) from the Strait of Messina (central Mediterranean Sea). Marine Biology Research 10, 927–933.

Bearzi, G., Reeves, R., Remonato, E., Pierantonio, N. and Airoldi, S. (2011) Risso’s dolphin, Grampus griseus, in the Mediterranean Sea. Mammalian Biology-Zeitschrift für Säugetierkunde 76, 385–400.

Bello, G. (1991) Role of cephalopods in the diet of the swordfish, Xiphias gladius, from the eastern Mediterranean Sea. Bulletin of Marine Science 49, 312–324.

Berthon, J. (1987) Relations trophiques entre quelques espèces d’échinodermes et le phytobenthos dans la baie de Port-Cros (Var, France).

Beverthon, R. and Holt, S. (1957) On the dynamics of exploited fish populations. Fisheries Investigations 1, 1–533.

Blanco, C., Salomón, O. and Raga, J.A. (2001) Diet of the bottlenose dolphin (Tursiops truncatus) in the western Mediterranean Sea. Journal of the Marine Biological Association of the United Kingdom 81, 1053–1058.

Boudaya, L., Neifar, L., Taktak, A., Ghorbel, M. and Bouain, A. (2007) Diet of Chelidonichthys obscurus and Chelidonichthys lastoviza (Pisces: Triglidae) from the Gulf of Gabes (Tunisia). Journal of Applied Ichthyology 23, 646–653.

Boyle, P.R. (1990) Cephalopod biology in the fisheries context. Fisheries Research 8, 303–321.

Bradai, M., Ghorbel, M., Jarboui, O. and Bouain, A. (1998) Feeding habits of Spondyliosoma cantharus, Diplodus puntazzo and Diplodus Vulgaris (Teleostei, Sparidae) in the Gulf of Gabes Tunisia. 35.

Bradai, M., Saidi, B. and Enajjar, S. (2012) Elasmobranchs of the Mediterranean and Black sea: status, ecology and biology. Bibliographic analysis. Studies and Reviews. Studies and Reviiew 91.

Bradai, M.N. and Bouain, A. (1990) Régime alimentaire de Scorpaena porcus et de S.scrofa (Teleostei, Scorpenidae), du golfe de Gabes, Tunisie. Cybium 14, 207–216.

Brando, V.E., Ceccarelli, R., Libralato, S. and Ravagnan, G. (2004) Assessment of environmental management effects in a shallow water basin using mass-balance models. Ecological Modelling 172, 213–232.

Butler, M., Bollens, S.M., Burkhalter, B., Madin, L.P. and Horgan, E. (2001) Mesopelagic fishes of the Arabian Sea: Distribution, abundance and diet of Chauliodus pammelas, Chauliodus sloani, Stomias affinis, and Stomias nebulosus. Deep-Sea Research Part II: Topical Studies in Oceanography 48, 1369–1383.

Canepa, A., Fuentes, V., Sabatés, A., Piraino, S., Boero, F. and Gili, J.M. (2014) Pelagia noctiluca in the mediterranean sea. In: Jellyfish Blooms, Vol. 9789400770. Springer Netherlands, Dordrecht, pp 237–266.

Canese, S., Cardinali, A., Fortuna, C.M., Giusti, M., Lauriano, G., Salvati, E. and Greco, S. (2006) The first identified winter feeding ground of fin whales (Balaenoptera physalus) in the Mediterranean Sea. Journal of the Marine Biological Association of the United Kingdom 86, 903–907.

Cannizzaro, L., Garofalo, G., Levi, D., Rizzo, P. and Gancitano, S. (1995) Raja clavata in the Sicilian Channel. Biologia Marina Mediterranea 2, 257–267.

Capapé, C. (1976) Etude du régime alimentaire de l’Aigle de mer, Myliobatis aquila (L., 1758) des côtes tunisiennes. ICES Journal of Marine Science 37, 29–35.

Capapé, C. (1977) Etude du regime alimentaire de la Mourine vachette, Pteromylaeus bovinus (Geoffroy Saint-Hilaire, 1817) (Pisces, Myliobatidae) des cotes tunisiennes. ICES Journal of Marine Science 37, 214–220.

Capapè, C. (1975) Etude du régime alimentaire de Squallus bainvillei (Risso, 1826) des cotes tunisienne. Bull. Inst. Natl. Sci. Tech. Océanogr. 4, 61–73.

Capapé, C. and Azouz, A. (1976) Etude du régime alimentaire de deux Raies communes dans le golfe de Tunis: Raja miraletus LINNÉ, 1758 et R. radula, DELAROCHE, 1809. Rapp. Comm. int Mer Médit. 23, 246–249.

Carpentieri, P., Colloca, F. and Ardizzone, G. (2007) Rhythms of feeding activity and food consumption of two Mediterranean burrowing fishes: Gnathophis mystax (Delaroche) and Chlopsis bicolor Rafinesque. Marine Ecology 28, 487–495.

Carpentieri, P., Serpetti, N., Colloca, F., Criscoli, A. and Ardizzone, G. (2016) Food preferences and rhythms of feeding activity of two co-existing demersal fish, the longspine snipefish, Macroramphosus scolopax (Linnaeus, 1758), and the boarfish Capros aper (Linnaeus, 1758), on the Mediterranean deep shelf. Marine Ecology 37, 106–118.

Cartes, J.E. (1993) Diet of Deep-sea Brachyuran Crabs in the Western Mediterranean Sea. Marine Biology 117, 449–457.

Cartes, J.E. (1995) Diets of, and trophic resources exploited by, bathyal penaeoidean shrimps from the western Mediterranean. Marine & Freshwater Research 46, 889–996.

Cartes, J.E. (1994) Influence of depth and season on the diet of the deep-water aristeid Aristeus antennatus along the continental slope (400 to 2300 m) in the Catalan Sea (western Mediterranean). Marine Biology 120, 639–648.

Casale, P., Mazaris, A.D., Freggi, D., Vallini, C. and Argano, R. (2009) Growth rates and age at adult size of loggerhead sea turtles (Caretta caretta) in the Mediterranean Sea, estimated through capture-mark-recapture records. Scientia Marina 73, 589–595.

Catalán, I.A., Folkvord, A., Palomera, I., Quílez-Badía, G., Kallianoti, F., Tselepides, A. and Kallianotis, A. (2010) Growth and feeding patterns of European anchovy (Engraulis encrasicolus) early life stages in the Aegean Sea (NE Mediterranean). Estuarine, Coastal and Shelf Science 86, 299–312.

Chérif, M., Ben Amor, M.M., Selmi, S., Gharbi, H., Missaoui, H. and Capapé, C. (2011) Food and feeding habits of the red mullet, Mullus barbatus (actinopterygii: Perciformes: Mullidae), off the Northern Tunisian coast (central Mediterranean). Acta Ichthyologica et Piscatoria 41, 109–116.

Coll, M., Palomera, I. and Tudela, S. (2009) Decadal changes in a NW Mediterranean Sea food web in relation to fishing exploitation. Ecological Modelling 220, 2088–2102.

Coll, M., Palomera, I., Tudela, S. and Dowd, M. (2008) Food-web dynamics in the South Catalan Sea ecosystem (NW Mediterranean) for 1978-2003. Ecological Modelling 217, 95–116.

Coll, M., Palomera, I., Tudela, S. and Sardà, F. (2006) Trophic flows, ecosystem structure and fishing impacts in the South Catalan Sea, Northwestern Mediterranean. Journal of Marine Systems 59, 63–96.

Coll, M., Santojanni, A., Palomera, I., Tudela, S. and Arneri, E. (2007) An ecological model of the Northern and Central Adriatic Sea: Analysis of ecosystem structure and fishing impacts. Journal of Marine Systems 67, 119–154.

Colloca, F., Cardinale, M. and Ardizzone, G.D. (1997) Biology, spatial distribution and population dynamics of Lepidotrigla cavillone (Pisces: Triglidae) in the Central Tyrrhenian Sea. Fisheries Research 32, 21–32.

Coma, R., Gili, J. and Zabala, M. (1995) Trophic ecology of a benthic marine hydroid , Campanularia everta. Marine Ecology Progress Series 119, 211–220.

Costalago, D. and Palomera, I. (2014) Feeding of European pilchard (Sardina pilchardus) in the northwestern Mediterranean: from late larvae to adults. Scientia Marina 78, 41–54.

Cotté, C., Guinet, C., Taupier-Letage, I., Mate, B. and Petiau, E. (2009) Scale-dependent habitat use by a large free-ranging predator, the Mediterranean fin whale. Deep-Sea Research Part I: Oceanographic Research Papers 56, 801–811.

Cristo, M. (1998) Feeding ecology of Nephrops norvegicus (Decapoda: Nephropidae). Journal of Natural History 32, 1493–1498.

Derbal, F. and Kara, M. (2008) Composition du régime alimentaire du bogue Boops boops (Sparidae) dans le golfe d’Annaba (Algérie). Cybium 32, 325–333.

Duarte, C.M. and Cebrián, J. (1996) The fate of marine autotrophic production. Limnology and Oceanography 41, 1758–1766.

Duarte, C.M. and Chiscano, C.L. (1999) Seagrass biomass and production: A reassessment. Aquatic Botany 65, 159–174.

Ebert, D.A. and Bizzarro, J.J. (2007) Standardized diet compositions and trophic levels of skates (Chondrichthyes: Rajiformes: Rajoidei ). Developments in Environmental Biology of Fishes 80, 221–237.

Esposito, V., Battaglia, P., Castriota, L., Finoia, M.G., Scotti, G. and Andaloro, F. (2009) Diet of Atlantic lizardfish, Synodus saurus (Linnaeus, 1758) (Pisces: Synodontidae) in the central Mediterranean Sea. Scientia Marina 73, 369–376.

Fabi, G., Panfili, M., Spagnolo, A., Marittima, P. and Fiera, L. (1998) Note on feeding of Sciaena umbra l. (Osteichthyes: Sciaenidae) in the central Adriatic Sea. Rapp. Comm. int Mer Médit. 35, 426–427.

Fanelli, E., Badalamenti, F., D’Anna, G. and Pipitone, C. (2009a) Diet and trophic level of scaldfish Arnoglossus laterna in the southern Tyrrhenian Sea (western Mediterranean): Contrasting trawled versus untrawled areas. Journal of the Marine Biological Association of the United Kingdom 89, 817–828.

Fanelli, E., Badalamenti, F., D’Anna, G., Pipitone, C., Riginella, E. and Azzurro, E. (2011) Food partitioning and diet temporal variation in two coexisting sparids, Pagellus erythrinus and Pagellus acarne. Journal of Fish Biology 78, 869–900.

Fanelli, E. and Cartes, J.E. (2010) Temporal variations in the feeding habits and trophic levels of the three deep-sea demersal fishes from the western Mediterranean sea, based on stomach contents and stable isotope analysis. Marine Ecology Progress Series 402, 213–232.

Fanelli, E., Cartes, J.E., Badalamenti, F., Rumolo, P. and Sprovieri, M. (2009b) Trophodynamics of suprabenthic fauna on coastal muddy bottoms of the southern Tyrrhenian Sea (western Mediterranean). Journal of Sea Research 61, 174–187.

Fanelli, E., Rey, J., Torres, P. and Gil De Sola, L. (2009c) Feeding habits of blackmouth catshark Galeus melastomus Rafinesque, 1810 and velvet belly lantern shark Etmopterus spinax (Linnaeus, 1758) in the western Mediterranean. In: Journal of Applied Ichthyology, Vol. 25. Wiley/Blackwell (10.1111), pp 83–93.

Fasola, M., Bogliani, G., Saino, N. and Canova, L. (1989) Foraging, feeding and time‐activity niches of eight species of breeding seabirds in the coastal wetlands of the adriatic sea. Bolletino di zoologia 56, 61–72.

Fauchald, K. and Jumars, P. (1979) The diet of worms: a study of polychaete feeding guilds. Oceanography and Marine Biology. An Annual Review 17, 193–284.

Filiz, H. and Bilge, G. (2004) Length-weight relationships of 24 fish species from the North Aegean Sea, Turkey. Journal of Applied Ichthyology 20, 431–432.

Filiz, H. and Toğulga, M. (2009) Age and growth, reproduction and diet of the black goby, (Gobius niger) from Aegean Sea, Turkey. Journal of FisheriesSciences.com 3, 243–265.

Follesa, M.C., Mulas, A., Cabiddu, S., Porcu, C., Deiana, A.M. and Cau, A. (2010) Diet and feeding habits of two skate species, Raja brachyura and Raja miraletus (Chondrichthyes, Rajidae) in Sardinian waters (centralwestern Mediterranean). Italian Journal of Zoology 77, 53–60.

Frantzis, A., Berthon, J. and Maggiore, F. (1988) Relations trophiques entre les oursins Arbacia lixula et Paracentrotus lividus et le phytobenthos infralittoral dans la baie de Port-Cros. Sci. Report Port-Cros Nat. Park 14, 81–140.

Froglia, C. and Giannini, S. (1989) Field observations on diel rhythms in catchability and feeding of Squilla mantis (L.) (Crustacea, Stomatopoda) in the Adriatic sea. In: in: E.A. Ferrero (ed.), Biology of Stomatopods. Selected Symposia and Monographs U.Z.I., Vol. 3. Mucchi, pp 221–228.

Gharbi, H. and Ktari, M.H. (1979) Regime alimentaire des rougets (Mullus barbatus L., 1758 et M. surmuletus L., 1758) du Golfe de Tunis. Bull. Inst. natn. scient. tech. Ocean. Peche Salammbo 6, 41–52.

Goñi, R., Quetglas, A. and Reñones, O. (2001) Diet of the spiny lobster Palinurus elephas (Decapoda: Palinuridea) from the Columbretes Islands Marine Reserve (north-western Mediterranean). Journal of the Marine Biological Association of the United Kingdom 81, 347–348.

Greze, I.I. (1968) Feeding habits and food requirements of some amphipods in the Black Sea. Marine Biology 1, 316–321.

Gristina, M., Badalamenti, F., Barbera, G., D’Anna, G. and Pipitone, C. (1992) First data on the feeding habits of Aristeomorpha foliacea (RISSO) in the Sicilian Channel. Oebalia XII, 143–144.

Guénette, S. and Morato, T. (2001) The Azores Archipelago, 1997. In: Fisheries Impacts on North Atlantic Ecosystems: Models and Analyses. (ed D. In: Guénette, S., Christensen, V., Pauly). Fisheries Centre, University of British Columbia, pp 167–181.

Guerra, A. (1978) Sobre la alimentación y el comportamiento de Octopus vulgaris. Inv. Pesq. 42, 351–364.

Hattab, T., Ben Rais Lasram, F., Albouy, C., et al. (2013) An ecosystem model of an exploited southern Mediterranean shelf region (Gulf of Gabes, Tunisia) and a comparison with other Mediterranean ecosystem model properties. Journal of Marine Systems 128, 159–174.

Hattour, A. (2000) Contribution a l’étude des poissons pélagiques des eaux tunisiennes.

Innes, S., Lavigne, D.M., Earle, W.M. and Kovacs, K.M. (1987) Feeding Rates of Seals and Whales. The Journal of Animal Ecology 56, 115.

Ismen, A., Arslan, M., Yigin, C.C. and Bozbay, N.A. (2013) Age, growth, reproduction and feeding of John Dory, Zeus faber (Pisces: Zeidae), in the Saros Bay (North Aegean Sea). Journal of Applied Ichthyology 29, 125–131.

Jardas, I., Šantić, M. and Pallaoro, A. (2004) Diet composition and feeding intensity of horse mackerel, Trachurus trachurus (Osteichthyes: Carangidae) in the eastern Adriatic. Marine Biology 144, 1051–1056.

Kadri, H., Saïdi, B., Marouani, S., Bradai, M.N. and Bouaïn, A. (2013) Food habits of the rough ray Raja radula (Chondrichthyes: Rajidae) from the Gulf of Gabès (central Mediterranean Sea). Italian Journal of Zoology 80, 52–59.

Kapiris, K. (2004) Feeding ecology of Parapenaeus longirostris (Lucas , 1846) (Decapoda : Penaeidae) from the Ionian Sea ( Central and Eastern Mediterranean Sea ). Scientia Marina 68, 247–256.

Kitsos, M.S., Tzomos, T., Anagnostopoulou, L. and Koukouras, A. (2008) Diet composition of the seahorses, Hippocampus guttulatus Cuvier, 1829 and Hippocampus hippocampus (L., 1758) (Teleostei, Syngnathidae) in the Aegean Sea. Journal of Fish Biology 72, 1259–1267.

Lelli, S., Belluscio, A., Carpentieri, P. and Colloca, F. (2005) Ecologia trofica di Ilex Coindetti e Todaropsis eblanae (Cephalopoda:Ommastrephidae) nel Mar Tirreno centrale. Biologia Marina Mediterranea 12, 531–534.

Lindley, J.A. (1982) Continuous plankton records: Geographical variations in numerical abundance, biomass and production of euphausiids in the North Atlantic Ocean and the North Sea. Marine Biology 71, 7–10.

Lipari, R., Badalamenti, F. and D’Anna, G. (1998) Relazioni trofiche e selezione alimentare di Mullus barbatus L. (1758) nella comunità a sabbie fini del Golfo di Castellammare (Sicilia N/O). Biol. Mar. Medit. 5, 513–516.

Lomiri, S., Scacco, U., Mostarda, E. and Andaloro, F. (2008) Size-related and temporal variation in the diet of the round sardinella, Sardinella aurita (Valenciennes, 1847), in the central Mediterranean Sea. Journal of Applied Ichthyology 24, 539–545.

Mackinson, S. and Daskalov, G. (2007) An ecosystem model of the North Sea to support an ecosystem approach to fisheries management: description and parameterisation. Science Series Technical Report 142, 196 pp.

Macpherson, E. (1979) Estudio sobre el regimen alimentario de algunos peces en el Mediterráneo Occidental. Misc. Zool. 5, 93–107.

Madurell, T. and Cartes, J.E. (2005) Trophodynamics of a deep-sea demersal fish assemblage from the bathyal eastern Ionian Sea (Mediterranean Sea). Deep Sea Research Part I: Oceanographic Research Papers 52, 2049–2064.

Marabello, F., Guglielmo, L., Granata, Antonia and Sidoti, O. (1996) Studi preliminari sulle abitudini alimentari di Todarodes sagittatus (Cephalopoda) nel Tirreno meridionale. In: Atti dell’11°Congresso dell’Associazione Italiana di Oceanologia e Limnologia. pp 271–278.

Martinho, F., Sá, C., Falcão, J., Cabral, H.N. and Pardal, M. a (2012) Comparative feeding ecology of two elasmobranch species, Squalus blainvile and Scyliorhinus canicula, off the coast of Portugal. Fishery Bulletin 110, 71–84.

Massutì, E., Deudero, S., Sanchez, P. and MoralesNin, B. (1998) Diet and feeding of dolphin (Coryphaena hippurus) in western Mediterranean waters. Bulletin of Marine Science 63(2), 329–341.

Matallanas, J. (1982) Notes on the feeding habits of Epigonus denticulatus (Pisces, Apogonidae) in the Catalan sea (Western Mediterranean). Vie Milieu 32, 77–81.

Maynou, F. and Cartes, J.E. (1998) Daily ration estimates and comparative study of food consumption in nine species of deep-water decapod crustaceans of the NW Mediterranean. Marine Ecology Progress Series 171, 221–231.

Menif, D. (2000) Les Gobiidés des côtes tunisiennes : Morphologie et biologie de Zosterisessor ophiocephalus (Pallas, 1811) et Gobius niger Linnaeus, 1758.

Mertz, G. and Myers, R.A. (1998) A simplified formulation for fish production. Canadian Journal of Fisheries and Aquatic Sciences 55, 478–484.

Milisenda, G., Rosa, S., Fuentes, V.L., Boero, F., Guglielmo, L., Purcell, J.E. and Piraino, S. (2014) Jellyfish as prey: Frequency of predation and selective foraging of Boops boops (vertebrata, actinopterygii) on the mauve stinger pelagia noctiluca (cnidaria, scyphozoa). PLoS ONE 9, e94600.

Molinero, A. and Flos, R. (1991) Influence of sex and age on the feeding habits of the common sole Solea solea. Marine Biology 111, 493–501.

Morat, F. (2007) Régime alimentaire de la population de cormoran huppé de Méditerranée (Phalacrocorax aristotelis desmarestii) de Riou. Marseilles: CEEP-DIMAR, Centre d’Océanologie 2007. 23pp., 23.

Moreno, T. and Castro, J.J. (1995) Community structure of the juvenile of coastal pelagic fish species in the Canary Islands waters. Scientia Marina 59, 405–413.

Morote, E., Olivar, M.P., Villate, F. and Uriarte, I. (2008) Diet of round sardinella, Sardinella aurita, larvae in relation to plankton availability in the NW Mediterranean. Journal of Plankton Research 30, 807–816.

Morte, S., Redon, M. and Sanz-Brau, A. (2001) Diet of Scorpaena porcus and Scorpaena notata (Pisces: Scorpaenidae) in the western Mediterranean. Cah. Biol. Mar. 42, 333–344.

Morte, S., Redon, M.J. and Sanz-Brau, A. (1999) Feeding habits of Trachinus draco off the eastern coast of Spain (western Mediterranean). Vie et Milieu 49, 287–291.

Moutopoulos, D.K., Libralato, S., Solidoro, C. and Stergiou, K.I. (2013) Toward an ecosystem approach to fisheries in the Mediterranean Sea: Multi-gear/multi-species implications from an ecosystem model of the Greek Ionian Sea. Journal of Marine Systems 113–114, 13–28.

Mulas, A., Bellodi, A., Cannas, R., et al. (2015) Diet and feeding behaviour of longnosed skate Dipturus oxyrinchus. Journal of Fish Biology 86, 121–138.

Nagy, K.A. (1987) Field metabolic rate and food requirement scaling in mammals and birds. Ecological Monographs 57, 111–128.

Najai, S. and Ktari, M.H. (1974) Etude du régime alimentaire de la seiche commune Sepia officinalis Linné, 1758 (Mollusque, Céphalopode) du golfe de Tunis. Hull. hist. nam. scient, tech. Océnogr. Pêche Salammbô 6, 53–61.

Navarro, J., Coll, M., Preminger, M. and Palomera, I. (2013) Feeding ecology and trophic position of a Mediterranean endemic ray: Consistency between sexes, maturity stages and seasons. Environmental Biology of Fishes 96, 1315–1328.

Opitz, S. (1996) Trophic interactions in Caribbean coral reefs, ICLARM. International Center for Living Aquatic Resources Management, Manila.

Ouannes-Ghorbel, A., Jarboui, O., Bradai, M. and Bouain, A. (2005) Régime alimentaire de Symphodus (crenilabrus) cinereus (Bonnaterre, 1788) des côtes de la région du golfe de Gabès (Tunisie). Bull. Mus. Hist. Nat. Marseille 61, 17–22.

Pauly, D. (1980) On the interrelationships between natural mortality, growth parameters, and mean environmental temperature in 175 fish stocks. ICES Journal of Marine Science 39, 175–192.

Pauly, D., Trites, A.W., Capuli, E. and Christensen, V. (1998) Diet composition and trophic levels of marine mammals. In: ICES Journal of Marine Science, Vol. 55. Oxford University Press, pp 467–481.

Pearson, M. and Gage, J.D. (1984) Diets of some deep-sea brittle stars in the Rockall Trough. Marine Biology 82, 247–258.

Pepe, P., Badalamenti, F. and D’Anna, G. (1998) Abitudini alimentari di Diplodus sargus nell’area delle strutture artificiali di Alcamo Marina (Golfo di Castellammare, Sicilia nord-occidentale. Biologia Marina Mediterranea 5, 367–370.

Pepe, P., Badalamenti, F. and D’Anna, G. (1996) Abitudini alimentari di Diplodus vulgaris sulle strutture artificiali del Golfo di Castellammare (Sicilia nord-occidentale). Biologia Marina Mediterranea 3, 514–515.

Pierce, G.J., Boyle, P.R., Hastie, L.C. and Santos, M.B. (1994) Diets of squid Loligo forbesi and Loligo vulgaris in the northeast Atlantic. Fisheries Research 21, 149–163.

Pinnegar, J.K. and Polunin, N.V.C. (2000) Contributions of stable-isotope data to elucidating food webs of Mediterranean rocky littoral fishes. Oecologia 122, 399–409.

Pipitone, C. (1987) Etologia alimentare di Mullus surmuletus (L.) sui fondali di Vergine Maria, golfo di Palermo.

Piroddi, C., Bearzi, G., Gonzalvo, J. and Christensen, V. (2011) From common to rare: The case of the Mediterranean common dolphin. Biological Conservation 144, 2490–2498.

Piroddi, C., Coll, M., Steenbeek, J., Moy, D.M. and Christensen, V. (2015) Modelling the Mediterranean marine ecosystem as a whole: Addressing the challenge of complexity. Marine Ecology Progress Series 533, 47–65.

Piroddi, C., Giovanni, B. and Villy, C. (2010) Effects of local fisheries and ocean productivity on the northeastern Ionian Sea ecosystem. Ecological Modelling 221, 1526–1544.

Plounevez, S. and Champalbert, G. (2000) Diet, feeding behaviour and trophic activity of the anchovy (Engraulis encrasicolus L.) in the Gulf of Lions (Mediterranean Sea). Oceanologica Acta 23, 175–192.

Polunin, N.V.C., Morales-Nin, B., Pawsey, W.E., Cartes, J.E., Pinnegar, J.K. and Moranta, J. (2001) Feeding relationships in Mediterranean bathyal assemblages elucidated by stable nitrogen and carbon isotope data. Marine Ecology Progress Series 220, 13–23.

Quetglas, A., González, M., Carbonell, A. and Sánchez, P. (2001) Biology of the deep-sea octopus Bathypolypus sponsalis (Cephalopoda: Octopodidae) from the western Mediterranean Sea. Marine Biology 138, 785–792.

Reeves, R. and Notarbartolo di Sciara, G. (2006) The Status and Distribution of Cetaceans in the Black Sea and Mediterranean Sea: Workshop Report-Monaco 5-7 March 2006.

Ribes, M., Coma, R. and Gili, J.M. (1999) Natural diet and grazing rate of the temperate sponge Dysidea avara (Demospongiae, Dendroceratida) throughout an annual cycle. Marine Ecology Progress Series 176, 179–190.

Ristow, D., Feldmann, F., Scharlau, W. and Wink, M. (1990) Population structure, philopatry and mortality of Cory’s Shearwater Calonectris d. diomedea. Die Vogelwelt 111, 172–181.

Romano, C., Fanelli, E., D’Anna, G., Pipitone, C., Vizzini, S., Mazzola, A. and Badalamenti, F. (2016) Spatial variability of soft-bottom macrobenthic communities in northern Sicily (Western Mediterranean): Contrasting trawled vs. untrawled areas. Marine Environmental Research 122, 113–125.

Rosas-Luis, R., Villanueva, R. and Sánchez, P. (2014) Trophic habits of the Ommastrephid squid Illex coindetii and Todarodes sagittatus in the northwestern Mediterranean Sea. Fisheries Research 152, 21–28.

Rumolo, P., Basilone, G., Fanelli, E., et al. (2017) Linking spatial distribution and feeding behavior of Atlantic horse mackerel (Trachurus trachurus) in the Strait of Sicily (Central Mediterranean Sea). Journal of Sea Research 121, 47–58.

Rumolo, P., Bonanno, A., Barra, M., et al. (2016) Spatial variations in feeding habits and trophic levels of two small pelagic fish species in the central Mediterranean Sea. Marine Environmental Research 115, 65–77.

Russo, G., Gianguzza, P. and Zava, B. (1999) Osservazioni sulla dieta di Caretta caretta (Linnaeus, 1758) in Mediterraneo. Biol. Mar. Medit. 6, 602–604.

Sabatés, A., Pagès, F., Atienza, D., Fuentes, V., Purcell, J.E. and Gili, J.M. (2010) Planktonic cnidarian distribution and feeding of Pelagia noctiluca in the NW Mediterranean Sea. Hydrobiologia 645, 153–165.

Ben Salem, M. (1988) Régime alimentaire de Trachurus trachurus (Linnaeus, 1758) et de T. mediterraneus (Steindachner, 1868), (poissons, téléostéens, Carangidae) de la province atlantico méditerranéenne. Cybium 12, 247–253.

Šantić, M., Jardas, I. and Pallaoro, A. (2004) Diet composition and feeding intensity of Mediterranean horse mackerel, Trachurus mediterraneus (Osteichthyes: Carangidae), in the central Adriatic Sea. Acta Adriatica 45, 43–50.

Šantić, M., Paladin, A. and Agović, A. (2011) Diet of common stingray, Dasyatis pastinaca (Chondrichthyes: Dasyatidae) in the eastern Adriatic Sea. Cahiers de Biologie Marine 52, 349–356.

Santos, M.B., Clarke, M.R. and Pierce, G.J. (2001) Assessing the importance of cephalopods in the diets of marine mammals and other top predators: Problems and solutions. Fisheries Research 52, 121–139.

Sarà, G. and Sarà, R. (2007) Feeding habits and trophic levels of bluefin tuna Thunnus thynnus of different size classes in the Mediterranean Sea. Journal of Applied Ichthyology 23, 122–127.

Sarà, M. (1993) Feeding habits of Cory’s shearwater (Calonectris diomedea) in the central Mediterranean Sea." Status and conservation of seabirds. SEO/BirdLife and Medmaravis, Madrid, 213–220.

Sever, T.M., Bayhan, B. and Taskavak, E. (2005) A Preliminary Study on the Feeding Regime of European Pilchard (Sardina pilchardus Walbaum 1792) in Izmir Bay, Turkey, Eastern Aegean Sea. NADA, WorldFish Center Quaterly 28, 41–48.

Sever, T.M., Filiz, H., Bayhan, B., Taskavak, E. and Bilge, G. (2008) Food habits of the hollowsnout grenadier, Caelorinchus caelorhincus (Risso, 1810), in the Aegean Sea, Turkey. Belgian Journal of Zoology 138, 81–84.

Šifner, S.K. and Vrgoč, N. (2009) Diet and feeding of the musky octopus, Eledone moschata, in the northern adriatic sea. Journal of the Marine Biological Association of the United Kingdom 89, 413–419.

Sinopoli, M., Fanelli, E., D’Anna, G., Badalamenti, F. and Pipitone, C. (2012) Assessing the effects of a trawling ban on diet and trophic level of hake, Merluccius merluccius, in the southern Tyrrhenian Sea. Scientia Marina 76, 677–690.

Sinopoli, M., Pipitone, C., Campagnuolo, S., Campo, D., Castriota, L., Mostarda, E. and Andaloro, F. (2004) Diet of young-of-the-year bluefin tuna, Thunnus thynnus (Linnaeus, 1758), in the southern Tyrrhenian (Mediterranean) Sea. Journal of Applied Ichthyology 20, 310–313.

Stagioni, M., Montanini, S. and Vallisneri, M. (2013) Feeding habits of anglerfish, Lophius budegassa (Spinola, 1807) in the Adriatic Sea, north-eastern Mediterranean. Journal of Applied Ichthyology 29, 374–380.

Stefanescu, C. and Cartes, J. (1992) Benthopelagic habits of adult specimens of Lampanyctus crocodilus (Risso, 1810)(Osteichthyes, Myctophidae) in the western Mediterranean deep slope. Scientia Marina 56, 69–74.

Stergiou, K. and Karpouzi, V.S. (2002) Feeding habits and trophic levels of Mediterranean fish. Reviews in Fish Biology and Fisheries 11, 217–254.

Tecchio, S., Coll, M., Christensen, V., Company, J.B., Ramírez-Llodra, E. and Sardà, F. (2013) Food web structure and vulnerability of a deep-sea ecosystem in the NW Mediterranean Sea. Deep-Sea Research Part I: Oceanographic Research Papers 75, 1–15.

Trites, A.W. and Pauly, D. (1998) Estimating mean body masses of marine mammals from maximum body lengths. Canadian Journal of Zoology 76, 886–896.

Tsarin, S. (1994) Age, growth, and some production characteristics of Ceratoscopelus warmingii (Myctophidae) in the tropical zone of the Indian Ocean. Journal of Ichthyology c/c of Voprosy Ikhtiologii 34, 59.

Tudela, S. and Palomera, I. (1995) Diel feeding intensity and daily ration in the anchovy Engraulis encrasicolus in the northwest Mediterranean Sea during the spawning period. Marine Ecology Progress Series 129, 55–61.

UNEP-MAP-RAC/SPA (2014) Seabird status and conservation in the Sicily Channel / Tunisian Plateau. By C. Carboneras. Draft internal report for the purposes of the Mediterranean Regional Workshop to Facilitate the Description of Ecologically or Biologically Significant Marine Areas, Malaga, Spain, 7–11 April 2014.

Valls, M., Quetglas, A., Ordines, F. and Moranta, J. (2011) Feeding ecology of demersal elasmobranchs from the shelf and slope off the Balearic Sea (western Mediterranean). Scientia Marina 75, 633–639.

Vannucci, S., Mancusi, C., Serena, F., Cuoco, C. and Voliani, A. (2006) Feeding ecology of rays in the southern Ligurian Sea. Biol. Mar. Medit. 13, 296–297.

Vasilakopoulos, P., Pavlidis, M. and Tserpes, G. (2011) On the diet and reproduction of the oilfish Ruvettus pretiosus (Perciformes: Gempylidae) in the eastern Mediterranean. Journal of the Marine Biological Association of the United Kingdom 91, 873–881.

Zghidi, W., Ezzeddine-Najai, S., Charfi-Cheikhrouha, F. and El Abed, A. (2003) Régime alimentaire du poulpe commun Octopus vulgaris Cuvier, 1797. Marine Life 13, 45–52.
