# Supplementary material for: Benthic-pelagic coupling mediates interactions in Mediterranean mixed fisheries: An ecosystem modeling approach
Source: PLoS One. 2019 Jan 15;14(1):e0210659. doi: 10.1371/journal.pone.0210659 (PMC6333361; doi:10.1371/journal.pone.0210659)
Supplement: S2 Table — For each functional group (FG) are detailed inputs: biomass (B; t km−2), production/biomass ratio (P/B; yr−1), consumption/biomass ratio (Q/B; yr−1); Landing and Discards are expressed in t km−2 year−1. Outputs: trophic level (TL), ecotrophic efficiency (EE; for most of the FG except for BO, EUP, MB), production/consumption (P/Q), respiration/assimilation (R/A), omnivory index (OI). Dom = Domain: p = pelagic, d = demersal, b = benthic. (DOCX) [file pone.0210659.s003.docx]

**S2 Table**.

| N° | Dom | FG | TL | B (tkm^-^²) | P/B(year^-1^) | Q/B(year^-1^) | EE | P/Q | R/A | Landings | Discards | OI |
| --- | --- | --- | --- | --- | --- | --- | --- | --- | --- | --- | --- | --- |
| 1 | p | SB | **3.58** | 2.8E-05 | 4.48 | 79.17 | **0.000** | **0.057** | **0.929** | 0.0E+00 | 0.0E+00 | **0.87** |
| 2 | p | MM | **4.35** | 3.7E-02 | 0.06 | 11.48 | **0.000** | **0.005** | **0.993** | 0.0E+00 | 0.0E+00 | **0.32** |
| 3 | p | TUR | **3.25** | 2.5E-02 | 0.16 | 2.60 | **0.000** | **0.062** | **0.923** | 0.0E+00 | 0.0E+00 | **0.29** |
| 4 | p | XIP | **4.51** | 5.9E-03 | 0.62 | 5.00 | **0.318** | **0.125** | **0.844** | 2.4E-02 | 1.8E-03 | **0.15** |
| 5 | p | THU | **4.56** | 4.9E-02 | 0.52 | 5.00 | **0.432** | **0.104** | **0.870** | 1.1E-02 | 3.4E-04 | **1.91** |
| 6 | p | LPL | **4.47** | 2.4E-01 | 0.40 | 5.00 | **0.262** | **0.080** | **0.900** | 2.4E-02 | 1.5E-03 | **0.10** |
| 7 | p | MPL | **4.02** | 6.4E-02 | 1.29 | 7.46 | **0.498** | **0.174** | **0.783** | 7.4E-03 | 8.5E-05 | **0.20** |
| 8 | p | SPL | **3.37** | 1.6E-01 | 1.29 | 6.76 | **0.982** | **0.191** | **0.761** | 5.1E-03 | 3.7E-05 | **0.09** |
| 9 | d | HAK0 | **3.48** | 1.1E-03 | 5.00 | 70.49 | **0.237** | **0.071** | **0.91** | 0.0E+00 | 0.0E+00 | **0.02** |
| 10 | d | HAK1 | **3.62** | 4.1E-03 | 3.00 | 30.25 | **0.509** | **0.099** | **0.88** | 2.3E-04 | 3.1E-05 | **0.02** |
| 11 | d | HAK2 | **4.08** | 1.4E-02 | 1.43 | 15.37 | **0.916** | **0.093** | **0.88** | 7.0E-04 | 2.3E-05 | **0.18** |
| 12 | d | HAK3 | **4.31** | 2.2E-02 | 1.56 | 8.61 | **0.915** | **0.181** | **0.77** | 2.1E-02 | 6.9E-04 | **0.13** |
| 13 | d | HAK4 | **4.49** | 2.1E-03 | 1.01 | 4.67 | **0.972** | **0.216** | **0.73** | 1.9E-03 | 6.2E-05 | **0.06** |
| 14 | d | MUL0 | **2.90** | 2.6E-03 | 5.00 | 38.99 | **0.522** | **0.128** | **0.84** | 0.0E+00 | 0.0E+00 | **0.39** |
| 15 | d | MUL1 | **2.90** | 1.4E-02 | 3.20 | 15.41 | **0.656** | **0.252** | **0.69** | 0.0E+00 | 0.0E+00 | **0.37** |
| 16 | d | MUL2 | **3.30** | 4.0E-02 | 1.50 | 7.00 | **0.620** | **0.214** | **0.73** | 4.9E-03 | 2.2E-04 | **0.04** |
| 17 | d | MUL3 | **3.36** | 2.0E-02 | 2.10 | 4.32 | **0.677** | **0.426** | **0.47** | 1.0E-02 | 4.4E-04 | **0.10** |
| 18 | d | TRA | **3.56** | 8.2E-01 | 1.20 | 3.62 | **0.869** | **0.332** | **0.59** | 1.2E-02 | 5.2E-03 | **0.43** |
| 19 | d | PAG | **3.39** | 2.4E-02 | 0.90 | 4.50 | **0.523** | **0.200** | **0.75** | 7.4E-03 | 4.7E-05 | **0.27** |
| 20 | d | DFS | **3.61** | 3.0E-01 | 1.14 | 3.60 | **0.975** | **0.317** | **0.6** | 7.5E-03 | 8.5E-04 | **0.50** |
| 21 | d | DFH | **2.93** | 1.3E+00 | 1.82 | 8.03 | **0.948** | **0.227** | **0.72** | 7.0E-03 | 1.5E-03 | **0.54** |
| 22 | d | DSM | **2.89** | 1.4E+00 | 1.22 | 4.72 | **0.955** | **0.258** | **0.68** | 3.7E-02 | 1.7E-03 | **0.49** |
| 23 | d | DSP | **3.93** | 1.3E-01 | 0.75 | 3.25 | **0.544** | **0.230** | **0.71** | 8.4E-03 | 6.6E-04 | **0.40** |
| 24 | d | DSR | **3.13** | 2.0E-01 | 1.11 | 4.93 | **0.585** | **0.225** | **0.72** | 1.1E-02 | 1.2E-03 | **0.43** |
| 25 | d | MSC | **2.88** | 1.9E+00 | 2.39 | 10.21 | **0.659** | **0.234** | **0.71** | 0.0E+00 | 0.0E+00 | **0.43** |
| 26 | d | MSG | **3.31** | 4.8E-01 | 0.99 | 4.50 | **0.358** | **0.220** | **0.73** | 0.0E+00 | 0.0E+00 | **0.47** |
| 27 | d | MSP | **3.79** | 3.3E-01 | 1.00 | 4.33 | **0.860** | **0.231** | **0.71** | 0.0E+00 | 0.0E+00 | **0.04** |
| 28 | d | RSH | **3.51** | 3.1E-01 | 0.65 | 2.60 | **0.014** | **0.248** | **0.69** | 2.3E-03 | 6.8E-05 | **0.33** |
| 29 | d | RSS | **3.42** | 2.3E-02 | 0.49 | 2.91 | **0.076** | **0.169** | **0.79** | 8.1E-04 | 3.3E-05 | **0.16** |
| 30 | d | SSH | **3.92** | 1.6E-01 | 0.72 | 3.39 | **0.154** | **0.212** | **0.73** | 8.9E-04 | 2.3E-04 | **0.24** |
| 31 | d | SSS | **3.97** | 6.9E-02 | 1.02 | 4.61 | **0.434** | **0.222** | **0.72** | 6.0E-04 | 2.0E-05 | **0.36** |
| 32 | p | ENG | **3.36** | 5.6E-01 | 1.90 | 13.50 | **0.598** | **0.140** | **0.82** | 5.0E-02 | 3.8E-03 | **0.08** |
| 33 | p | SAR | **3.30** | 6.6E-01 | 1.00 | 8.40 | **0.557** | **0.119** | **0.85** | 2.1E-02 | 8.6E-04 | **0.06** |
| 34 | p | EPI | **2.58** | 4.5E+00 | 1.70 | 7.20 | **0.444** | **0.236** | **0.7** | 7.5E-03 | 3.3E-04 | **0.48** |
| 35 | d | CEBH | **3.25** | 3.2E-01 | 2.53 | 12.27 | **0.602** | **0.207** | **0.74** | 3.8E-02 | 9.6E-04 | **0.28** |
| 36 | d | CEBS | **3.55** | 3.5E-01 | 3.13 | 12.25 | **0.238** | **0.255** | **0.68** | 0.0E+00 | 0.0E+00 | **0.14** |
| 37 | d | CEPH | **3.63** | 4.2E-02 | 8.74 | 24.88 | **0.917** | **0.351** | **0.56** | 4.1E-03 | 3.9E-05 | **0.51** |
| 38 | d | CEPS | **3.91** | 1.3E-01 | 4.20 | 13.06 | **0.781** | **0.322** | **0.6** | 7.7E-03 | 7.6E-05 | **0.45** |
| 39 | d | DNS | **2.81** | 1.2E+00 | 3.37 | 12.54 | **0.690** | **0.269** | **0.66** | 1.6E-03 | 1.1E-05 | **0.64** |
| 40 | d | DNH | **2.44** | 4.6E-01 | 2.26 | 11.67 | **0.999** | **0.194** | **0.76** | 1.5E-04 | 1.0E-06 | **0.40** |
| 41 | d | DRS | **2.78** | 1.0E+00 | 1.62 | 7.40 | **0.889** | **0.219** | **0.73** | 5.4E-03 | 3.6E-05 | **0.53** |
| 42 | d | DRH | **2.44** | 2.5E-01 | 1.72 | 8.72 | **0.814** | **0.191** | **0.76** | 3.6E-03 | 2.6E-04 | **0.36** |
| 43 | d | ARF | **3.09** | 1.6E-01 | 1.30 | 7.32 | **0.180** | **0.178** | **0.78** | 1.4E-02 | 2.5E-03 | **0.29** |
| 44 | d | PWL | **3.31** | 1.5E-01 | 1.34 | 8.00 | **0.822** | **0.168** | **0.79** | 1.1E-01 | 1.9E-02 | **0.17** |
| 45 | b | SUP | **2.31** | 4.3E+00 | 7.73 | 36.51 | **0.800** | **0.212** | **0.74** | 1.1E-03 | 2.9E-02 | **0.23** |
| 46 | b | O | **2.22** | 1.8E+00 | 4.92 | 19.61 | **0.798** | **0.251** | **0.69** | 9.3E-04 | 1.0E-01 | **0.20** |
| 47 | b | FF | **2.00** | 7.4E-01 | 4.44 | 18.15 | **0.797** | **0.245** | **0.69** | 0.0E+00 | 5.6E-02 | **0.00** |
| 48 | b | DF | **2.05** | 4.6E+00 | 2.88 | 10.60 | **0.733** | **0.272** | **0.66** | 0.0E+00 | 4.3E-03 | **0.05** |
| 49 | b | C | **2.51** | 5.7E-01 | 6.11 | 17.44 | **0.901** | **0.350** | **0.56** | 0.0E+00 | 5.4E-04 | **0.30** |
| 50 | b | PAR | **2.65** | 1.3E-02 | 8.00 | 30.99 | **0.876** | **0.258** | **0.68** | 0.0E+00 | 0.0E+00 | **0.34** |
| 51 | b | SCA | **2.57** | 6.3E-02 | 6.50 | 31.04 | **0.918** | **0.209** | **0.74** | 0.0E+00 | 3.2E-05 | **0.34** |
| 52 | b | H | **2.13** | 1.4E-03 | 5.08 | 24.20 | **0.679** | **0.210** | **0.74** | 0.0E+00 | 0.0E+00 | **0.11** |
| 53 | b | GRA | **2.57** | 7.0E-03 | 8.44 | 39.99 | **0.966** | **0.211** | **0.74** | 0.0E+00 | 9.3E-03 | **0.34** |
| 54 | b | SF | **2.00** | 3.5E-02 | 6.35 | 30.48 | **0.345** | **0.208** | **0.74** | 0.0E+00 | 4.2E-03 | **0.00** |
| 55 | b | PF | **2.00** | 7.3E-04 | 3.03 | 11.31 | **0.938** | **0.268** | **0.66** | 0.0E+00 | 5.6E-04 | **0.00** |
| 56 | b | BO | **2.52** | **2.1E-01** | 9.00 | 33.00 | 0.950 | **0.273** | **0.66** | 0.0E+00 | 0.0E+00 | **0.28** |
| 57 | p | EUP | **2.59** | **1.0E+00** | 6.00 | 30.00 | 0.950 | **0.200** | **0.75** | 0.0E+00 | 0.0E+00 | **0.52** |
| 58 | p | ZG | **2.84** | 8.5E-02 | 18.45 | 48.80 | **0.974** | **0.378** | **0.53** | 0.0E+00 | 4.7E-05 | **0.58** |
| 59 | p | ZL | **2.77** | 2.4E-01 | 30.00 | 102.00 | **0.883** | **0.294** | **0.63** | 0.0E+00 | 0.0E+00 | **0.51** |
| 60 | p | ZM | **2.26** | 8.7E-01 | 28.55 | 107.46 | **0.773** | **0.266** | **0.61** | 0.0E+00 | 0.0E+00 | **0.30** |
| 61 | p | ZS | **2.53** | 3.7E+00 | 15.63 | 80.73 | **0.799** | **0.194** | **0.71** | 0.0E+00 | 0.0E+00 | **0.28** |
| 62 | p | PB | **2.00** | 6.0E+00 | 25.87 | 89.78 | **0.983** | **0.288** | **0.66** | 0.0E+00 | 0.0E+00 |  |
| 63 | b | BB | **2.00** | 2.1E+00 | 25.87 | 89.78 | **0.976** | **0.288** | **0.66** | 0.0E+00 | 0.0E+00 |  |
| 64 | p | PS | **1.00** | 2.9E+00 | 205.62 | 0.00 | **0.209** |  |  | 0.0E+00 | 0.0E+00 |  |
| 65 | p | DFL | **1.00** | 4.8E-02 | 220.08 | 0.00 | **0.990** |  |  | 0.0E+00 | 0.0E+00 |  |
| 66 | p | PL | **1.00** | 3.0E+00 | 131.49 | 0.00 | **0.259** |  |  | 0.0E+00 | 0.0E+00 |  |
| 67 | b | MB | **1.00** | **7.4E-01** | 30.00 | 0.00 | 0.900 |  |  | 0.0E+00 | 0.0E+00 |  |
| 68 | b | SG | **1.00** | 1.8E+01 | 5.47 | 0.00 | **0.091** |  |  | 0.0E+00 | 0.0E+00 |  |
| 69 | b | MA | **1.00** | 6.9E+00 | 5.00 | 0.00 | **0.401** |  |  | 0.0E+00 | 0.0E+00 |  |
| 70 | b | DC | **1.00** | 1.9E-01 |  |  | **0.876** |  |  | 0.0E+00 | 0.0E+00 |  |
| 71 | d | SPOM | **1.00** | 3.9E+00 |  |  | **0.995** |  |  | 0.0E+00 | 0.0E+00 |  |
| 72 | b | BD | **1.00** | 8.4E+01 |  |  | **1.000** |  |  | 0.0E+00 | 0.0E+00 |  |
