# Supplementary material for: Benthic-pelagic coupling mediates interactions in Mediterranean mixed fisheries: An ecosystem modeling approach
Source: PLoS One. 2019 Jan 15;14(1):e0210659. doi: 10.1371/journal.pone.0210659 (PMC6333361; doi:10.1371/journal.pone.0210659)
Supplement: S3 Table — Raw data across functional groups of predators in columns and preys in rows. (DOCX) [file pone.0210659.s004.docx]

S3_Table
