# Supplementary material for: Benthic-pelagic coupling mediates interactions in Mediterranean mixed fisheries: An ecosystem modeling approach
Source: PLoS One. 2019 Jan 15;14(1):e0210659. doi: 10.1371/journal.pone.0210659 (PMC6333361; doi:10.1371/journal.pone.0210659)
Supplement: S4 Table — B total biomass (exluding detritus), Q total consumption, Ex sum of all exports, R sum of all respiratory flow, FD sum of all flows into detritus, P sum of all productions, TST total system throughput, PP net primary production, TE mean transfer efficiency, C capacity, O overhead, A ascendency, FCI model stress, Y catches, TLm mean trophic level of catches, Y/PP gross efficiency. (DOCX) [file pone.0210659.s005.docx]

**S3 Table**.

| Main statistics | Units | Strait of Sicily | Gulf of Gabes | Catalan Sea | Adriatic Sea | Aegean Sea | Greek  Ionian Sea | Med Sea |
| --- | --- | --- | --- | --- | --- | --- | --- | --- |
| B | t km^-2^y^-1^ | 7.6E+01 | 7.4E+01 | 5.3E+01 | 1.3E+02 | 3.3E+01 | 2.1E+01 | 4.3E+01 |
| Q | t km^-2^y^-1^ | 1.6E+03 | 1.4E+03 | 5.9E+02 | 1.3E+03 | 8.7E+02 | 3.5E+02 | 9.2E+02 |
| Ex | t km^-2^y^-1^ | 6.9E-01 | 7.5E+02 | 1.3E+03 | 7.3E+02 | 2.7E+02 | 1.7E+00 | 1.3E+03 |
| R | t km^-2^y^-1^ | 8.6E+02 | 5.1E+02 | 2.3E+02 | 4.2E+02 | 2.7E+02 | 1.7E+02 | 2.9E+02 |
| FD | t km^-2^y^-1^ | 9.7E+02 | 1.1E+03 | 1.6E+03 | 1.4E+03 | 5.6E+02 | 5.1E+02 | 1.5E+03 |
| P | t km^-2^y^-1^ | 1.6E+03 | 1.8E+03 | 1.7E+03 | 1.6E+03 | 7.9E+02 | 6.0E+02 | - |
| TST | t km^-2^y^-1^ | 3.4E+03 | 3.8E+03 | 3.7E+03 | 3.8E+03 | 2.0E+03 | 1.0E+03 | 4.0E+03 |
| PP | t km^-2^y^-1^ | 1.2E+03 | 1.3E+03 | 1.3E+03 | 1.1E+03 | 5.4E+02 | 5.0E+02 | 1.6E+03 |
| TE | % | 1.9E+01 | 1.9E+01 | 1.3E+01 | 1.0E+01 | 1.7E+01 | 1.3E+01 | 9.2E+00 |
| Development |  |  |  |  |  |  |  |  |
| PP/R |  | 1.3E+00 | 2.5E+00 | 5.7E+00 | 2.7E+00 | 2.0E+00 | 2.8E+00 | 5.6E+00 |
| PP/B |  | 1.5E+01 | 1.7E+01 | 2.5E+01 | 8.8E+00 | 1.6E+01 | 2.3E+01 | 3.8E+01 |
| R/B |  | 1.1E+01 | 6.9E+00 | 4.3E+00 | 3.2E+00 | 8.2E+00 | 8.2E+00 | 6.8E+00 |
| B/TST |  | 2.2E-02 | 1.9E-02 | 1.4E-02 | 3.4E-02 | 1.7E-02 | 2.1E-02 | 1.1E-02 |
| PP/TST |  | 3.4E-01 | 3.3E-01 | 3.5E-01 | 3.0E-01 | 2.7E-01 | 4.8E-01 | 4.0E-01 |
| FD/TST |  | 2.8E-01 | 3.0E-01 | 4.2E-01 | 3.6E-01 | 2.8E-01 | 4.9E-01 | 3.7E-01 |
| Q/TST |  | 4.7E-01 | 3.7E-01 | 1.6E-01 | 3.4E-01 | 4.4E-01 | 3.4E-01 | 2.3E-01 |
| R/TST |  | 2.5E-01 | 1.3E-01 | 6.2E-02 | 1.1E-01 | 1.4E-01 | 1.7E-01 | 7.3E-02 |
| Ex/TST |  | 2.0E-04 | 2.0E-01 | 3.5E-01 | 1.9E-01 | 1.4E-01 | 1.6E-03 | 3.3E-01 |
| PP/P |  | 7.4E-01 | 7.0E-01 | 7.6E-01 | 7.3E-01 | 6.8E-01 | 8.3E-01 | - |
| C | flowbits | 2.0E+04 | 1.7E+04 | 1.0E+04 | 1.5E+04 | 9.2E+03 | 3.9E+03 | - |
| A/C | % | 2.6E+01 | 2.7E+01 | 2.6E+01 | 2.7E+01 | 2.2E+01 |  | - |
| O/C | % | 7.4E+01 | 7.3E+01 | 7.5E+01 | 7.3E+01 | 7.8E+01 | 7.6E+01 | - |
| FCI | %of TST | 7.6E+00 | 7.4E+00 | 5.2E+00 | 1.5E+01 | 1.5E+01 | 1.4E+01 | 5.0E+00 |
| Food web |  |  |  |  |  |  |  |  |
| CoI |  | 2.0E-01 | - | - | - | - | - | 1.0E-01 |
| SOI |  | 3.0E-01 | - | 2.0E-01 | 1.9E-01 | 1.8E-01 | 3.6E-01 | 2.7E-01 |
| Fishing |  |  |  |  |  |  |  |  |
| Tot_Y | t km^-2^y^-1^ | 7.0E-01 | 1.7E+00 | 5.2E+00 | 2.4E+00 | 2.4E+00 | 1.7E+00 | - |
| TLm |  | 3.1E+00 | 3.4E+00 | 3.1E+00 | 3.1E+00 | 3.5E+00 |  | 3.1E+00 |
| Y/PP |  | 6.2E-04 | 1.0E-03 | 3.0E-03 | 2.0E-03 | 4.0E-03 | 3.0E-03 | 2.6E-04 |
