# Supplementary figures and images for: Benthic-pelagic coupling mediates interactions in Mediterranean mixed fisheries: An ecosystem modeling approach
Source: PLoS One. 2019 Jan 15;14(1):e0210659. doi: 10.1371/journal.pone.0210659 (PMC6333361; doi:10.1371/journal.pone.0210659)

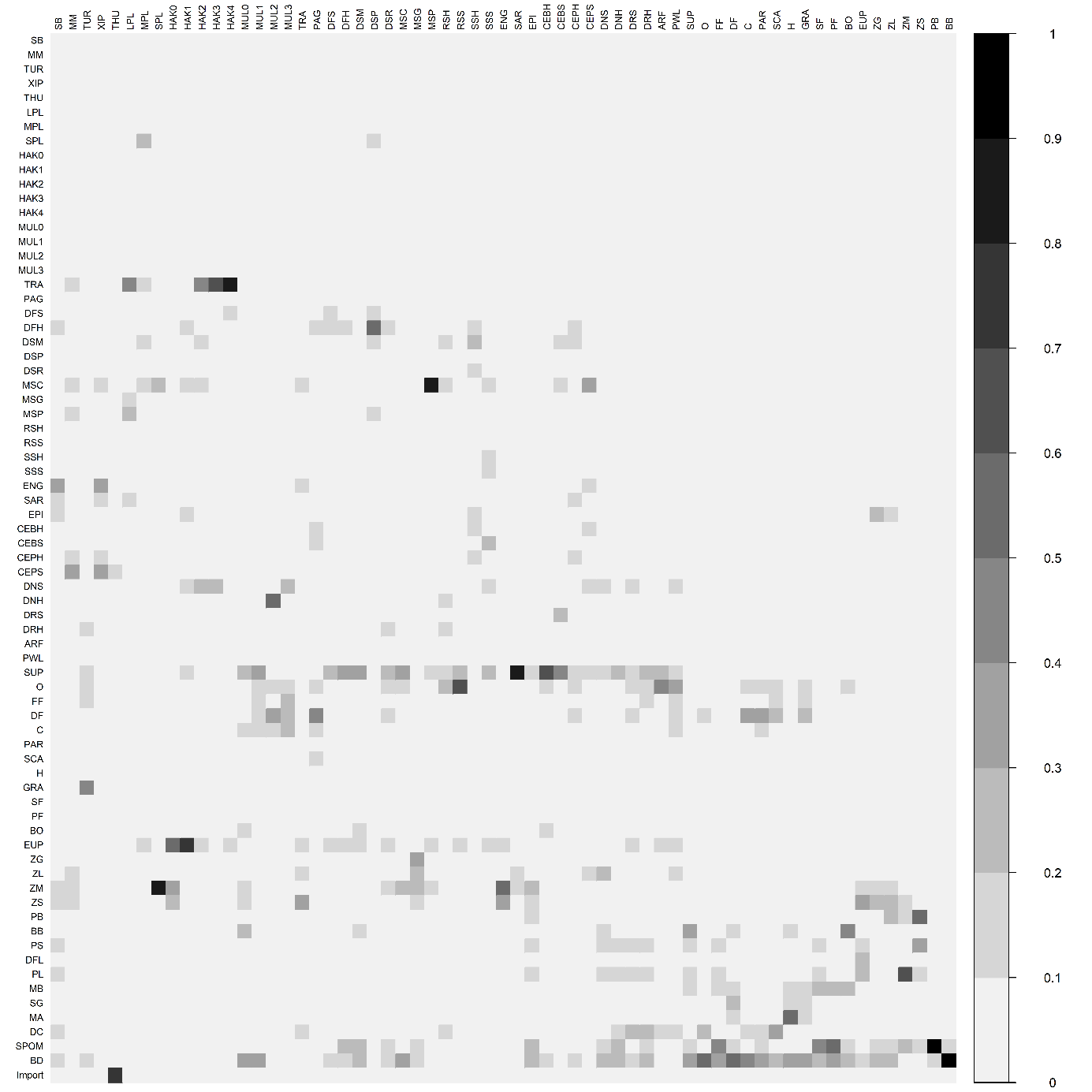

Supplement: S1 Fig — Proportion of preys (rows) in the diet of the predators (columns) in ranks. Fraction of import in the diet (fraction of energy assumed to be taken out of the system) are also indicated. (TIF) [file pone.0210659.s006.tif]

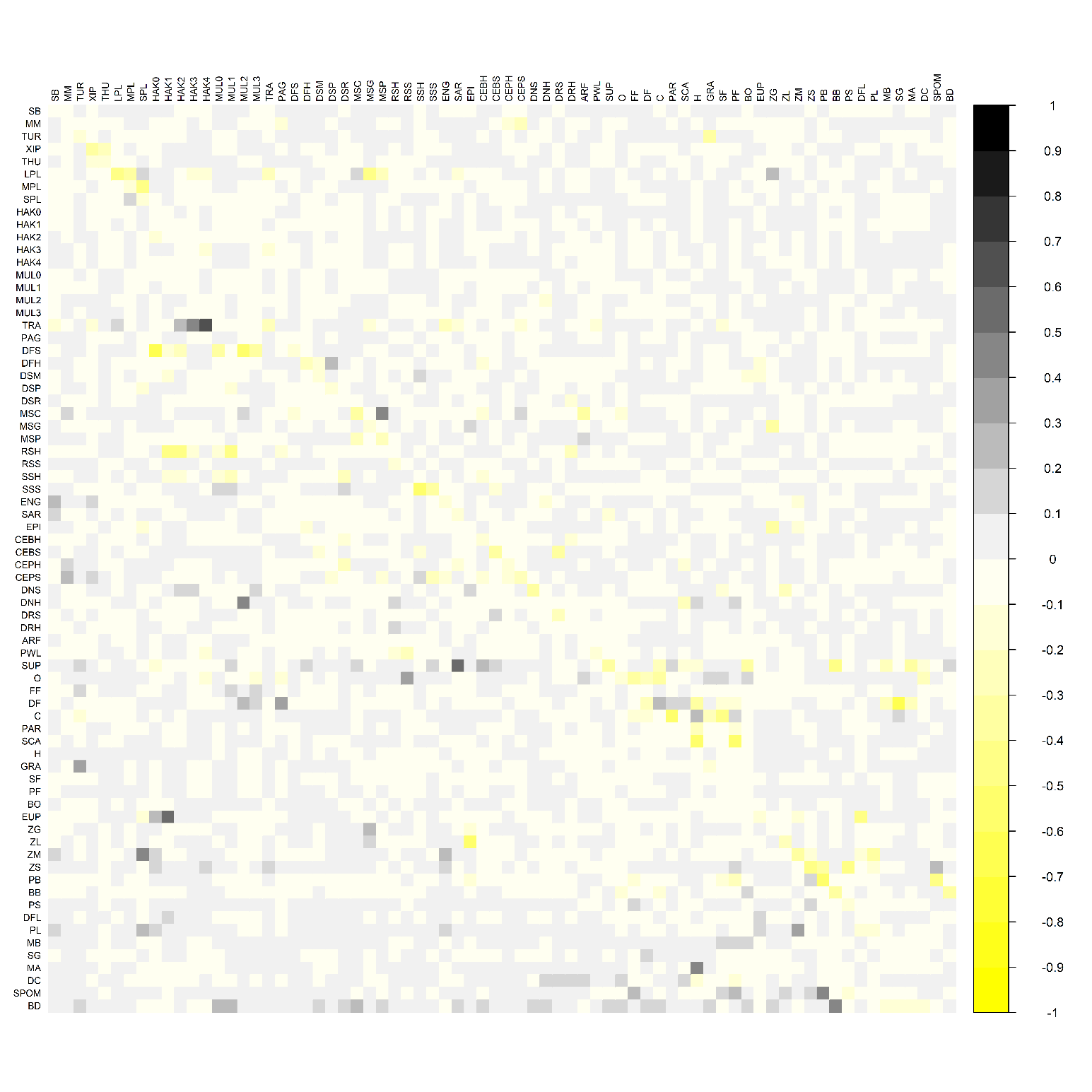

Supplement: S2 Fig — Rows are impacting FGs and columns impacted FGs. (TIF) [file pone.0210659.s007.tif]
